# Synergistic Photophysical and Mechanical Enhancement in Europium Supramolecular Hydrogel by Incorporating Gd(DPA)_3_ Complex (DPA = Dipicolinate)

**DOI:** 10.1002/open.70202

**Published:** 2026-04-23

**Authors:** Caroliny O. Cavalcante, Suelen C. F. Pereira, Sanderson H. S. Malta, José Y. R. Silva, Wilson B. Jr, Leonis Lourenço da Luz, Juliana A. B. da Silva, Severino Alves Júnior

**Affiliations:** ^1^ Departamento de Química Fundamental Universidade Federal de Pernambuco Cidade Universitária Recife Brasil; ^2^ Departamento de Física Universidade Federal de Pernambuco Cidade Universitária Recife Brasil; ^3^ Núcleo Interdisciplinar de Ciências Exatas e da Natureza Universidade Federal de Pernambuco Caruaru Brasil

**Keywords:** computational modeling; metallogel, gels, lanthanide, luminescence

## Abstract

Lanthanide hydrogels HGLn with iminodiacetic acid (Ln = Eu and Gd) and Na_3_[Ln(DPA)_3_]·9H_2_O complexes (DPA = dipicolininate) were successfully synthesized via hydrothermal microwave‐assisted and via precipitation, respectively. Incorporation of Ln(DPA)_3_ complexes into the HGLn hydrogels resulted in the improvement of the spectroscopic properties and mechanical behavior. Rheological studies of the europium hydrogels doped with Gd(DPA)_3_ complex (HGEu‐Gd) showed a significant increase in the stiffness of the material by increasing the concentration of the incorporated complex. This effect also influences the increase in luminescence intensity of the HGEu‐Gd hybrid. A computational modeling study of the interaction between the complex and hydrogel matrix suggests that this interaction does not affect the coordination symmetry of either Eu^3+^ or Gd^3+^. On the other hand, there were changes in the corresponding emission lifetimes, although not accompanied by changes in the emission spectral profile. The individual contributions to the luminescence of the hybrid gel were related to the maintenance of individual material characteristics and those resulting from hydrogel‐complex interactions.

## Graphical abstract

Lanthanide‐based hydrogels incorporating Ln(DPA)_3_ complexes are synthesized and exhibit enhanced mechanical and luminescent properties. Increased complex concentration improves stiffness and emission intensity without altering coordination symmetry. Computational insights reveal preserved structural features, while lifetime changes highlight hydrogel–complex interactions, offering tunable hybrid materials with improved performance and potential multifunctional applications.

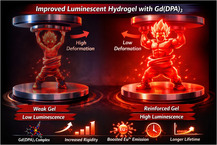

## 1 Introduction

Gels are soft materials with a wide range of applications, commonly found in our daily lives in cosmetics, medicines, soaps, and in the food industry. These diverse applications of gels can be attributed to their unique physical properties. Gels exhibit properties intermediate between the elasticity of solids and the viscosity of liquids [1, 2]. Furthermore, hydrogels (gels composed mainly of water) stand out from other subclasses of gels because they are biocompatible, biodegradable, low‐cost, and quickly obtainable [3].

Furthermore, in recent years there has been a growing interest in the investigation of supramolecular metallogels [4]. A metallogel is a gel that contains gelling agents and coordinated metal ions. The reason for the growing interest in this class of gels stems from the diversity of coordination modes between metals and ligands, thus facilitating control in the self‐assembly process during gel formation, in addition to bringing together properties related to metal ions such as magnetism and luminescence [5]. Despite the various advantages of using hydrogels, they usually exhibit limited mechanical behavior, with low elasticity. Thus, over the years, research groups have been making increasing efforts to synthesize hydrogels with better mechanical properties [6, 7, 8].

Metallo‐hydrogels based on trivalent lanthanide ions have incorporated luminescent and magnetic properties of these ions and can be applied in a wide range of applications [9, 10]. Multimetal metallogels have developed and presented luminescence responsive to multiple stimuli, including temperature, pH, ultrassound, and ion photobleaching [11, 12, 13]. For instance, Mahapatra et al. developed colorless lanthanide‐based supramolecular hydrogels with color emission dependent on the molar ratio of europium and terbium ions enabling the material to be used as invisible security ink. For instance, Mahapatra et al. developed colorless lanthanide‐based supramolecular hydrogels with color emission dependent on the molar ratio of europium and terbium ions anabling the material to be used as invisible security ink [14, 15]. Furthermore, these materials present rheological properties dependent to multistimuli, such as heating and mechanical stimuli [14], and pH‐responsive color change [15]. In another hand, Zhang et al. have developed a hydrogel formed by a poly‐ligand with Eu^3+^/Tb^3+^ mixed ions which showed white light emission, as well as applied as fingerprint imaging, colorful OLED, and response to acid and alkali vapors [16]. In previous reports, the amino acid iminodiacetic and the lanthanide ions have been used to develop luminescent metallo‐hydrogels with potential applications as light‐emitting diodes (LEDs), unique luminescent barcodes dependent on the excitation wavelength, and thin films for use in tamper seals, as well as drug loading. Also, their structural elucidation have been made through computational calculations [17, 18, 19].

From this perspective, advances in the incorporation of metal complexes into hydrogels have also been reported, with the aim of increasing the number of cross‐links, resulting in a mechanically stronger hydrogel, as well as conferring relevant luminescent properties, or improving the existing properties of gels already reported, for example, by controlling the intramolecular energy transfer process that leads to luminescence [20]. This approach allows for the design of materials with potential applications, for example, for monitoring drug release, temperature, pressure sensing etc. [21, 22].

Theoretical approaches play a fundamental role as a complement to experimental techniques, mainly in the context of investigating structural and photoluminescent properties of lanthanide‐based materials. These tools have been contributing to understanding the structural features, analysis, and assignment of spectroscopic signals, as well as to elucidating the energy transfer mechanisms involved in the luminescence processes. From geometries obtained by optimization in the ground state of suitable proposed models, it is possible to estimate relevant spectroscopic properties, including the energies of the singlet and triplet states, intensity parameters, and the energy transfer rates between the ligand and the lanthanide ion. This information is essential for understanding the energy transfer mechanism responsible for luminescent emission.

Given the scientific relevance of metallohydrogels, the objective of this work was to combine an experimental‐theoretical integrative approach to propose a multifunctional tunable platform with improved luminescent and mechanical properties and expanding the scope of its applications. For this, we integrate Na_3_[Ln(DPA)_3_]·9H_2_O complexes (DPA = dipicolinate anion; Ln = Eu^3+^ and Gd^3+^) into hydrogels based on lanthanide ions and iminodiacetic acid, HGLn (Ln = Eu^3+^ and Gd^3+^), and, from experimental evidence, we perform computational luminescent modeling of these systems to elucidate the main structural characteristics and understand the energy transfer mechanism that explains the observed luminescent properties.

## 2 Results and Discussion

### 2.1 Thermogravimetric Analysis of the Ln(DPA)_3_ (Ln = Eu^3+^ e Gd^3+^) Complexes

**FIGURE 1:**
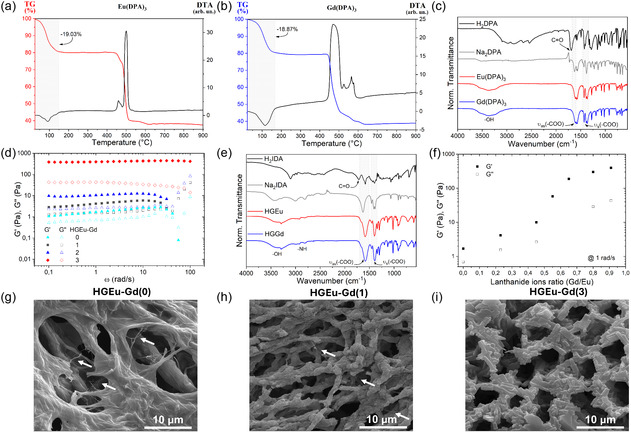
DTA and TGA curves of the (a) Eu(DPA)_3_ and (b) Gd(DPA)_3_ complexes. (c) FTIR spectra of H_2_DPA and the Eu(DPA)_3_ and Gd(DPA)_3_ complexes. (d) Oscillatory frequency sweep rheological measurements of the HGEu‐Gd(0) and HGEu‐Gd hybrid hydrogels, showing the storage modulus (G) and loss modulus (G) as a function of angular frequency (ω). (e) FTIR spectra of H_2_IDA and the HGEu and HGGd hydrogels. (f) From data of panel (d) at 1 rad/s, G’ and G’’ as function of ion ration of HGEu‐Gd hybrid hydrogels. SEM micrographs of the hydrogels (g) HGEu‐Gd(0), (h) HGEu‐Gd(1), and (i) HGEu‐Gd(3).

Ln(DPA)_3_ (Ln = Eu^3+^ e Gd^3+^) complexes were synthesized, and they were obtained as white powders. In order to estimate the number of crystallization water molecules, thereby determine the molar mass of each complex, differential thermal analysis (DTA) and thermogravimetric analysis (TG) were performed (Figure 1a,b). According to the DTA curves, the thermal events occurring up to 155°C are endothermic and attributed to the loss of nine crystallization and physiosorbed water molecules, corresponding to a mass loss of 19.03% (calculated: 18.45%) and 18.87% (calculated: 18.34%) for the Eu(DPA)_3_ and Gd(DPA)_3_ complexes, respectively. The compounds were stable up to 450°C and subsequently underwent decomposition of the dipicolinate ligand, which continued up to approximately 600°C through exothermic processes [23]. Above 600°C, a plateau was observed, corresponding to the presence of oxides, with residues of 37.50% (calculated: 37.27%) for 2/3Eu_2_O_3_ + 3/2Na_2_O, and 38.88% (calculated: 37.85%) for 2/3Gd_2_O_3_ + 3/2Na_2_O. Accordingly, the proposed molecular formulas for the obtained complexes are Na_3_[Eu(DPA)_3_].9(H_2_O) and Na_3_[Gd(DPA)_3_].9(H_2_O).

### 2.2 Infrared Analysis of the Ln(DPA)_3_ (Ln = Eu^3+^ e Gd^3+^) Complexes

Fourier‐transform infrared spectroscopy (FTIR) was employed to identify the functional groups present in the Eu(DPA)_3_ and Gd(DPA)_3_ complexes and to compare them with those of the H_2_DPA ligand and Na_2_DPA (Figure 1c). Broad and intense absorption bands observed in the spectra of Eu(DPA)_3_ and Gd(DPA)_3_ in the region of 3727–2904 cm^−1^ are attributed to O–H stretching vibrations of water molecules present in the structure, corroborating the TGA results. The characteristic C = O stretching band of H_2_DPA appeared at 1691 cm^−1^. For Na_2_DPA, the asymmetric ν_as_(COO^‐^) and symmetric ν_sym_(COO^‐^) stretching vibrations of the carboxylate groups were found at 1627 and 1380 cm^−1^, respectively. In the complexes, these bands shifted to 1607 and 1387 cm^−1^ for Eu(DPA)_3_ and at 1606 and 1385 cm^−1^ for Gd(DPA)_3_, indicating the coordination of the carboxylate groups to the metal centers [24]. The coordination mode was further analyzed by comparing the difference Δν (ν_as_ ‐ ν_sym_) between Na_2_DPA and the complexes. The Δν value for Na_2_DPA (247 cm^−1^), Eu(DPA)_3_ (220 cm^−1^), and Gd(DPA)_3_ (221 cm^−1^) are near due to Tridentate (O, N,O’‐chelate) coordination mode [25, 26].

### 2.3 Rheological Properties of the HGEu Gel

Lanthanide hydrogels prepared from iminodiacetic acid (H_2_IDA) and lanthanide oxide (HGLn; Ln = Eu^3+^ and Gd^3+^) were obtained as transparent and self‐supporting materials, as confirmed by the tube inversion test. Through rheological experiments, it is possible to study how deformation and flow occur in the synthesized hydrogels.

To evaluate the mechanical stability of the hydrogel network and to define safe experimental conditions for subsequent oscillatory measurements, a shear rate sweep test was performed on the as‐prepared HGEu hydrogel (Figure S14), in which the shear stress (σ) was monitored as a function of the applied shear rate (γ). The results show a marked deviation from linear stress growth above γ ≈ 0.4 s^−1^, indicating structural rupture of the gel network associated with irreversible disruption of the crosslinks [27]. Based on these observations, oscillatory rheological measurements were performed using a low strain amplitude (γ_0_ = 0.01), which is within the linear viscoelastic region and was selected for a conservative condition to ensure the preservation of the gel structure during frequency sweep experiments. In the applied frequency range, the G’ is nearly frequency‐independent up to 10 rad/s, and G′ exceeds G″ over the investigated frequency range up to values above 30 rad/s. This suggests that the HGEu‐Gd(0) is gelled with the formation of a strong gel network [27]. However, above 30 rad/s the HGEu gel lose their stability and G’ becomes larger than G’’ (Figure 1d).

### 2.4 Infrared Spectroscopy of the HGLn Gels

The amino acid H_2_IDA when associated with lanthanide ions in aqueous medium, generates hydrogels [17]. The resulting hydrogel exhibits noncovalent interactions, such as hydrogen bonding between IDA molecules, as well as Ln‐IDA coordination bonds [18, 19]. These interactions can be evidenced by FTIR analysis. The FTIR spectrum of H_2_IDA shows characteristic a band corresponding to the C = O stretching vibration at 1705 cm^−1^. The corresponding carbonyl signal in the hydrogel appears as asymmetric V_as_(COO^‐^) at 1585 cm^−1^, and symmetric V_sym_(COO^‐^) stretching vibrations at 1399 cm^−1^, observed for both HGEu and HGGd, indicating coordination between the metal ion and the ligand (Figure 1e). The two signals corresponding to V_sym_(COO^‐^) stretching are related to two coordination modes in which the IDA ligand interacts with the Ln ion [19]. To further elucidate the coordination mode of IDA ligand in the gel, a comparison with IR spectrum of the Na_2_IDA was made. The sodium salt exhibits ν_as_ and ν_sym_ bands at 1633 and 1409 cm^−1^, resulting in a Δν value of 224 cm^−1^. In contrast, the Δν values for HGEu and HGGd are 186 cm^−1^, indicating a chelating (bidentate)/bridging coordination mode of the carboxylate groups. Additionally, bands observed at 3293 and 2919 cm^−1^ are assigned to O–H and N–H stretching vibrations, respectively [17].

### 2.5 Rheological Properties of the Hybrid Gels HGLn‐Ln(x)

The viscoelastic behavior of HGEu‐Gd(1‐3) hybrids is similar to that of the pure HGEu‐Gd(0) gel (Figure 1d). The measurements were performed under low strain amplitude conditions, selected to preserve the structural integrity of the hydrogel network, as defined from the preliminary mechanical stability tests. For all samples, G′ exceeds G″ over the investigated frequency range up to values above 30 rad/s, such as HGEu‐Gd(0). However, only the most concentrated sample maintains its elasticity over the entire frequency interval. Also, a progressive increase in mechanical rigidity was observed, as evidenced by the systematic rise in the G’ and G’’ values. The remarkable progressive increase in the absolute values of G′ with increasing Gd(DPA)_3_ content reflects the reinforcement of the hydrogel network upon complex incorporation, and the systematic increase in the G’/G’’ ratio, with the incorporated complex increases, indicates a gradual enhancement of the elastic contribution to the viscoelastic response. Each module exhibits weak frequency dependence, indicating a mechanically stable network within the timescale probed by the oscillatory measurements. Figure 1f depicts values of G’ and G’’ extracted from Figure 1d, for a frequency of 1 rad/s, as function of the Gd/Eu ions ratio. One can notice the transition to a more robust gel occurring for ratios above 0.5. It is important to mention that the incorporation of just the DPA ligand destabilizes the gel, which becomes liquid.

### 2.6 Morphological Analysis

The morphologies of GEu‐Gd(0–3) gels were analyzed using scanning electron microscopy (SEM) images, after Lyophilization (Figure 1g‐i). The SEM micrograph of GEu‐Gd(0) xerogel confirmed the development of a cross‐linked fibrous network with fibers (Figure 1g). The white arrow points to nanometric fibers that form the gel network. The addition of the Gd(DPA)_3_ complex increases interaction between fibers, increasing the rigidity of the gel, and progressively leads to the formation of micrometric block‐like structures, more visible in the GEu‐Gd(3) xerogel (Figures 1h‐i).

### 2.7 Spectroscopic Properties of Ln(DPA)_3_ Complexes

The absorption spectrum of the Eu(DPA)_3_ and Gd(DPA)_3_ complexes in aqueous solution (Figure S1) displays an absorption band between 263 and 290 nm, corresponding to the π* ← π electronic transition in the aromatic ring of the dipicolinate anion [28]. The structure observed within the absorption band is attributed to the vibronic progressions.

Then, the Gd^3+^ ion allows for the investigation of the luminescent properties of the DPA ligand in the Gd(DPA)_3_ complex without interference of metal‐centered emission, since the emissive state of Gd^3+^ (^6^P_7/2_, 32,195 cm^−1^) lies at a higher energy than the emissive state of the dipicolinate anion (24,272 cm^−1^) [29].

**FIGURE 2:**
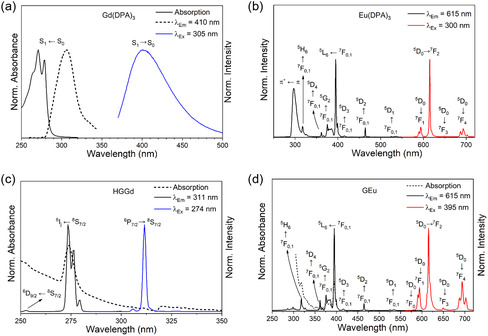
(a) Absorption, excitation (λ_Em_ = 400 nm) and emission (λ_Ex_ = 305 nm) spectra of Gd(DPA)_3_ in water. (b) Excitation (λ_Em_ = 615 nm) and emission (λ_Ex_ = 300 nm) spectra of Eu(DPA)_3_ in water. (c) Excitation (λ_Em_ = 311 nm) and emission (λ_Ex_ = 274 nm) spectra related to the IL band, of the HGGd hydrogel. (d) Absorption, excitation (λ_Em_ = 615 nm) and emission (λ_Ex_ = 395 nm) spectra of the HGEu hydrogel.

The excitation spectrum (λ_Em_ = 410 nm) of the Gd(DPA)_3_ complex shows a band with a maximum at 305 nm, associated with the S_1_ ← S_0_ transition of the dipicolinate ligand, (Figure 2a). The large shift between absorption and excitation spectra reflects the change in the molecular electron distribution for the excited state. On the other hand, the emission spectrum (λ_Ex_ = 305 nm) shows a broad band centered at 410 nm, related to fluorescence (S_1_ → S_0_) of the DPA ligand [30].

Such as Gd(DPA)_3_, the excitation spectrum (λ_Em_ = 615 nm) of the Eu(DPA)_3_ complex presents a wide band with maximum at 300 nm, corresponding to the π* ← π (S_0_ → S_1_) transition of the ligand, confirming its role as Eu^3+^ luminescence sensitizer (Figure 2b). In addition, narrow bands with maxima at 525, 464, 415, 395, 375, 361, and 317 nm correspond to the Eu^3+^ transitions ^5^D_1_←^7^F_0,1_, ^5^D_2_←^7^F_0,1_, ^5^D_3_←^7^F_0,1_, ^5^L_6_←^7^F_0,1_, ^5^G_J_←^7^F_0,1_, ^5^D_4_←^7^F_0,1_, and ^5^H_6_←^7^F_0,1_, respectively. The intensity of the ^5^L_6_ is greater than the ligand band due to the inner filter effect, due to the high absorbance of the DPA ligands at that concentration [31].

The emission spectrum exhibits narrow bands attributed to the ^5^D_0_→^7^F_J_ transition, with J = 1 – 4, having maxima at 594, 615, 648, and 693 nm, respectively. The hypersensitive ^5^D_0_→^7^F_2_ transition is the most intense (accounting for 61% of the integrated spectrum) and is mainly responsible for the characteristic red photoluminescence of the material [25]. The spectral profile with the number of Stark levels of 2 (^5^D_0_ → ^7^F_1_), 1 (^5^D_0_ → ^7^F_2_), and 3 (^5^D_0_ → ^7^F_4_) is compatible with a nine‐coordinate tricapped trigonal prism coordination polyhedron with site symmetry D_3h_ (Figure S2) [32, 33, 34].

The luminescence lifetime measurement for the Eu(DPA)_3_ complex (Figure S3) was carried out upon excitation at 300 nm and by monitoring emission at 615 nm, yielding a lifetime (τ) of 1.47 ms [35]. The decay curve was fitted using a mono‐exponential function, suggesting the presence of a single coordination environment for the Eu^3+^ ion in the material.

From the emission spectrum and the lifetime value, the radiative (A_rad_), and nonradiative (A_nrad_) decay rates, as well as the quantum efficiency (η), were determined and are presented in Table 1. According to the high η value of 35.7%, mainly influenced by the low nonradiative decay rate (A_nrad_), 437 s^−1^, the complex demonstrates efficient conversion of ultraviolet radiation into visible light. This value is near to the reported value of η = 41% for the Eu(DPA)_3_ complex [35].

**TABLE 1:** Intensity parameters (Ω_2_ and Ω_4_), radiative deactivation rate (Arad), nonradiative deactivation rate (Anrad), emission lifetime (τ), quantum efficiency ratios (η), and quantum yield (q).

System	**Ω**_**2**_ **(10** ^**−20**^ **cm** ^**2**^ **)**	**Ω**_**4**_ **(10** ^**−20**^ **cm** ^**2**^ **)**	**A**_**rad**_, **s** ^**−1**^	**A**_**nrad**_, **s** ^**−1**^	τ, ms	η, %	q, %
HGEu^exp.^	5.14	7.27	316	3132	0.29	9.2	—
HGEu^theo[a]^	5.14	7.27	314	3134	0.29	9.1	0.07
HGEu‐Gd(1)^exp.^	5.83	6.40	325	1907	0.45	14.6	—
HGEu‐Gd(2)^exp.^	6.79	5.63	342	1004	0.74	25.4	—
HGEu‐Gd(3)^exp.[c]^	6.68	5.64	341	459	1.25	42.6	—
HGEu‐Gd(3)^theo[b],[c]^	6.67	5.64	335	465	1.25	41.89	36.7

*Note:* Calculated for [a] [Eu_2_(IDA)_6_(H_2_O)_6_] complex model and for [b] [Eu_2_(IDA)_6_(HDPA)_2_(H_2_O)_4_]^2–^ complex model. [c] Lifetime related to the ligand state excitation.

### 2.8 Spectroscopic Properties of HGLn Hydrogels

The absorption spectrum of the HGGd complex shows narrow bands associated with f–f transitions overlapped by the onset of a broad absorption band attributed to an intraligand (IL) transition of the IDA ligand (Figure 2c) [18]. Due to the low molar absorptivity of this IL transition in the considered spectral region, narrow bands related to the Gd^3+^ ion at 252.5 nm (^6^D_9/2_ ← ^8^S_7/2_) and 274 nm (^6^I_J_ ← ^8^S_7/2_) are observed in the excitation spectrum. The emission spectrum of HGGd, acquired under excitation at 274 nm, exhibits narrow bands in the range of 300–320 nm, corresponding to transitions between split Stark levels of the ^6^P_7/2_ → ^8^S_7/2_ transition [36]. On the other hand, when the excitation is set at 305 nm, a broad band emission with a maximum at 420 nm is observed. Thus, the hydrogel HGGd exhibits dual‐band emission in both the ultraviolet and visible spectral regions, which can be switched through excitation wavelength.

The excitation spectrum of the HGEu hydrogel (Figure 2d) displays only sharp bands centered at 525, 464, 416, 395, 376, 361, and 318 nm, assigned to the Eu^3+^ transitions ^5^D_1_←^7^F_0,1_, ^5^D_2_←^7^F_0,1_, ^5^D_3_←^7^F_0,1_, ^5^L_6_←^7^F_0,1_, ^5^G_J_←^7^F_0,1_^5^D_4_←^7^F_0,1_, and ^5^H_6_←^7^F_0,1_, respectively, when monitored at 615 nm. This behavior indicates that the IDA ligand does not act as Eu^3+^ luminescence sensitizer. The emission spectrum (λ_Ex_ = 395 nm) displays the characteristic bands related to transitions ^5^D_0_→^7^F_J_ (J = 1 – 4) of the Eu^3+^ [19]. The high intensity of the ^5^D_0_→^7^F_2_ (forbidden transition by the electric dipole mechanism), relative to the ^5^D_0_→^7^F_1_ (allowed magnetic transition) along with the presence of the ^5^D_0_→^7^F_0_ (580 nm) transition, suggests that the Eu^3+^ ion is located at low‐symmetry environment without inversion center, possibly restricted to C_*n*_, C_nv_ or C_s_, Additionally, the absence of splitting in the ^5^D_0_→^7^F_0_ band indicates that there is only one luminescent symmetry site for the Eu^3+^ ion [32]. The luminescence decay curve of the HGEu hydrogel was fitted with a mono‐exponential function (Figure S4), with τ = 0.29 ms, corroborating the existence of a single coordination environment for the luminescent Eu^3+^ ions in this hydrogel [32, 37, 38].

### 2.9 Spectroscopic Properties of Hybrid Hydrogels HGLn‐Ln’

To understand the photoluminescent behavior of the hydrogels following the incorporation of the Ln(DPA)_3_ complexes and to correlate these findings with rheological results, hybrid of three different combination of lanthanide ions were prepared: (i) Gd(DPA)_3_ complex into HGEu, resulting in the hybrids HGEu‐Gd(x); (ii) Eu(DPA)_3_ complex into HGGd, resulting in the hybrids HGGd‐Eu(x); and (iii) Gd(DPA)_3_ complex into HGGd, resulting in the hybrids HGGd‐Gd(x), where x varies from 0 to 3, each designed for different investigative purposes.

The complex Gd(DPA)_3_ was used to improve the luminescence of the Eu^3+^ in the HGEu‐Gd(x) systems. To minimize any rehydration effect on the luminescence intensity of the HGEu, the HGEu‐Gd(0) was taken as a reference. Furthermore, the excitation spectrum of the rehydrated hydrogel, HGEu‐Gd(0), showed slight changes in the Stark components of the ^5^D_0_ → ^7^F_2_ (612 nm) and ^7^F_4_ (702 nm) transitions, suggesting small distortions in the Eu^3+^ coordination environment after the lyophilization and rehydration process (Figure S5). Thus, since all hybrid gels underwent rehydration, they were compared against the rehydrated HGLn‐Ln’(0) hydrogel.

The luminescence behavior of the EuDPA_3_ in aqueous solutions at concentration corresponding to the hybrids HGGd‐Eu(1), (2), and (3) was obtained. The small change in the intensity of the excitation band of the DPA ligand (Figure S6a) occurs due to the attenuation of the light beam along the optical path, caused by the high concentration of the complex. And thus, the emission spectra of the aqueous solutions, under excitation at the ligand (λ_Ex_ = 300 (1), 298 (2), and 296 (3) nm), exhibit similar spectral behavior (Figure S6b). On the other hand, the emission spectra obtained under direct Eu^3+^ excitation (λ_Ex_ = 395 nm) show a significant increase in emission intensity as the concentration increases. This behavior is due to the lower molar absorptivity coefficient of the lanthanide ion than that dipicolinate ligand and, thus, does not significantly attenuate the excitation been light at the optical path (Figure S6c) [35]. The image in Figure S6d shows the Eu(DPA)_3_ solution along the optical path with excitation at 302 nm, with a xenon lamp. The lifetimes for all concentrations displayed mono‐exponential decay, following excitation at both the Eu^3+^ ion and ligand (Table S1).

The excitation spectra of the HGEu‐Gd(1–3) hybrids, acquired by monitoring emission at 615 nm (^5^D_0_→^7^F_2_), show narrow absorption bands associated with the f–f transitions of the Eu^3+^ ion between 320 and 550 nm, as well as in HGEu‐Gd(0); however, a broad band attributed to S_*n*_ ← S_0_ transitions of the DPA ligand between 250 and 320 nm was observed (Figure S7a)**,** suggesting that DPA acts as a sensitizer for Eu^3+^ luminescence in the hybrid materials.

**FIGURE 3:**
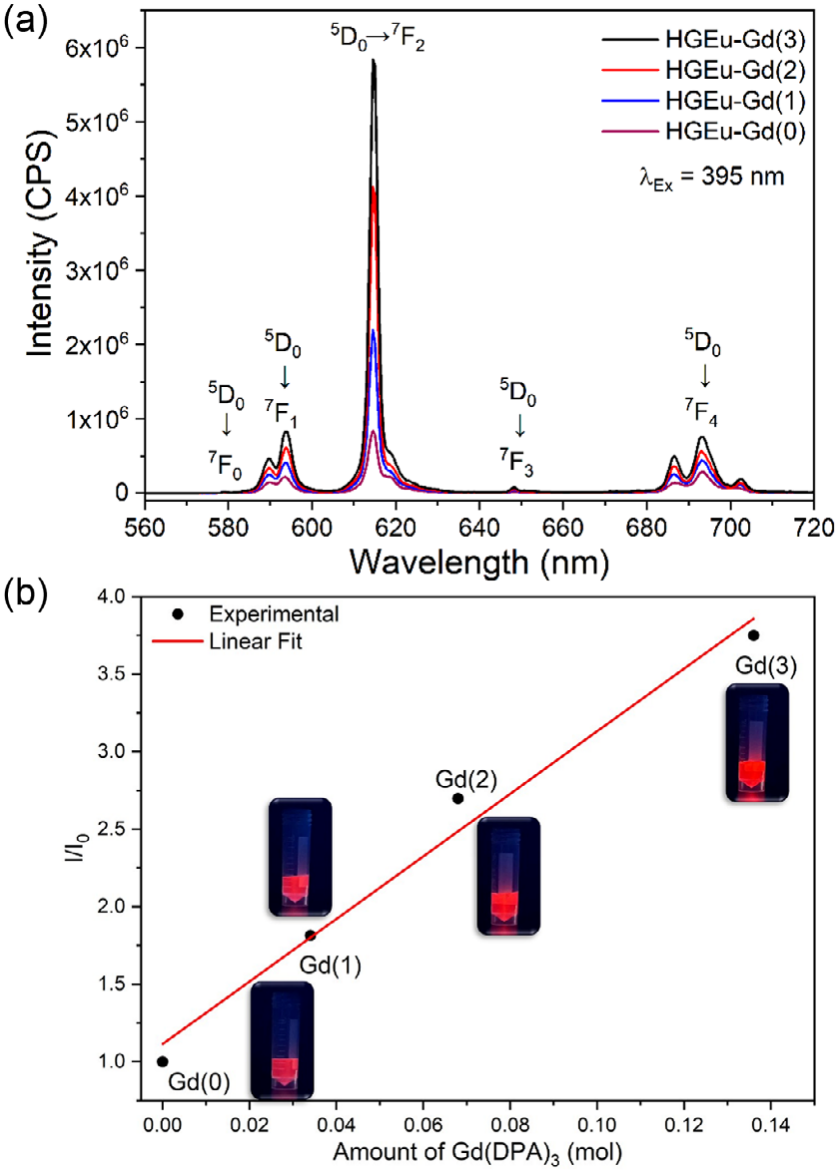
(a) Emission spectra (λ_Ex_ = 395 nm) of the HGEu‐Gd(0‐3) hybrids. (b) Plot of the ratio of the integrated intensity of the emission spectra (575 to 720 nm) of the HGEu‐Gd (1‐3), (I), and the integrated intensity of the rehydrated HGEu(0) (I_0_) versus amount of the Gd(DPA)_3_. along with the corresponding images obtained under excitation at 295 nm.

Since the Eu^3+^ ion concentration remains constant in the HGEu‐Gd(1)–(3) hydrogels, the unchanging intensity of the excitation band through the ligand in the hybrids (2) and (3) suggests that interactions with Eu^3+^ ions have reached saturation. However, the intensity of the f–f transitions of the Eu^3+^ ion is still increasing up to higher concentrations of the Gd(DPA)_3_ complex. As expected, the emission intensity of the hybrid hydrogels, upon excitation at both the ligand (λ_Ex_ = 296, 298 and 300 nm; Figure S7b) and directly at the Eu^3+^ ion (λ_Ex_ = 395 nm; Figure 3a), shows the same trends of excitation spectra. Furthermore, the emission spectral profiles are similar to those of the HGEu‐Gd(0) hydrogel, suggesting that interactions between the hydrogel network and the DPA ligand do not alter the coordination polyhedron geometry of the Eu^3+^ ion (Figure 3a) [33, 39].

The integrated intensity of the emission spectra (λ_Ex_ = 395 nm) for each hydrogel HGEu‐Gd(0‐3) (I) was normalized by the intensity of the rehydrated hydrogel (I_0_), and a plot of (I/I_0_) versus amount of Gd(DPA)_3_ complex has exhibited a linear increase (Figure 3b). Since the amount and concentration of Eu^3+^ ions are unchanged in all hydrogels, and the DPA ligand does not absorb in this spectral region (395 nm), there should be no enhancement in luminescence intensity under direct excitation of the ion. We hypothesize that this behavior results from two main interactions between the components of the hybrid: (i) interaction between the complex and the hydrogel network, which suppresses quenching pathways through increased rigidity of the system, and (ii) coordination of the DPA ligand to the Eu^3+^ ion within the gel fiber, thereby replacing quenchers from the first coordination sphere of the ion (OH oscillators from the coordinated water molecules) [8]. Photographic images of the HGEu–Gd(0)–(3) hydrogels under daylight and under UV irradiation are presented in Figure S7c. It is perceptible to the naked eye that, as the Gd(DPA)3 complex amount increases, the red emission intensities under both ligand and ion excitation.

In a similar study, Wang et al. demonstrated the improvement in the mechanical and luminescent properties of p(NIPAM‐co‐AA) (p(N‐isopropylacrylamide‐co‐acrylic acid)) based hydrogels by adding an Sm^3+^‐complex. They compared two Sm^3+^ complexes, both of which had 1,10‐phenanthroline and thenoyltrifluoroacetone as ligands; however, only one of them also had coordinated nitrate groups. The significant improvement in mechanical properties was observed only in the hydrogels in which the complex with NO_3_^–^ groups was inserted, and this was attributed to the interaction between the nitrates in the complex and carboxylic groups present in the hydrogels [6]. Li et al. also observed improved rheological and luminescent properties by incorporating lanthanide complexes with 1,6‐pyridinecarboxylic acid as polymer chain terminations in a hydrogel, where the complex units acted as junctions in the cross‐links of the polymer network [8].

The luminescence decay curves were obtained for HGEu‐Gd(1‐3), monitoring the emission intensity at 615 nm after excitation at both the emission center (395 nm, ^5^L_6_ ← ^7^F_0_ transition of Eu^3+^, Table S2) and the DPA ligand (Table 2). The hybrid hydrogels HGEu‐Gd(1‐3) exhibited bi‐exponential radiative decay. Since the fluorescence of Gd(DPA)_3_ does not occur in the same spectral region as the Eu^3+^ ion luminescence and the phosphorescence of the Gd(DPA)_3_ is not observed at room temperature, one lifetime component can be assigned to Eu^3+^ ion without direct influence with DPA ligand (not coordinated) and the other lifetime is related to Eu^3+^ under direct influence of the DPA ligand (coordinated). Thus, the shorter lifetime can be correlated with unmodified Eu^3+^ sites that retain the initial coordination environment of Eu^3+^ in the HGEu hydrogel, whereas the longer lifetime is associated with a fraction that is strongly influenced by interactions with the Gd(DPA)_3_ complex. Therefore, we propose that the Gd(DPA)_3_ complex interacts at the surface of the gel fibers, in which the supramolecular aggregated [Ln_2_(IDA)_6_(H_2_O)_6_] complex units of the HGEu gel [19] and the Gd(DPA)_3_ complex interacts to reinforce the supramolecular interaction between the HGEu fibers, and a DPA moiety coordinates to the Eu^3+^ replacing a water molecule acting as Eu^3+^‐luminescence sensitizer.

**TABLE 2:** Luminescence lifetime (τ_1_), pre‐exponential factors (A_1_), and average lifetime (τ_m_) obtained from exponential fitting of luminescence decay curves for the HGGd‐Eu(x) hybrids.

System	**λ**_**ex**_ **, nm**	**A**_**1**_ **, %**	τ_**1**_ **, ms**	τ_**2**_ **, ms**	τ_**m**_
**HGEu‐Gd (0)**	—	—	—	—	—
**HGEu‐Gd (1)**	296	39.8	0.26	60.2	0.95
**HGEu‐Gd (2)**	298	31.0	0.26	69.0	1.20
**HGEu‐Gd (3)**	301	18.6	0.21	81.4	1.25

To support this hypothesis, an additional experiment was performed: A small amount of Eu(NO_3_)_3_ was added to an aqueous solution of the Gd(DPA)_3_ 17 mmol L^−1^ to evaluate the possible action of the DPA ligand as sensitizer to Eu‐luminescence as well as the change in the emission spectral profile. As can be seen in Figure S8, after solubilization, the system became cloudy, and a white solid precipitate was observed. In addition, the emission spectral profile is quite different from that presented by Eu(DPA)_3_ complex, indicating that the Gd^3+^ has not been replaced by Eu^3+^; nevertheless, the broad and highly intense excitation band at 290 nm indicates that the DPA ligand acts as a sensitizer of europium luminescence. Therefore, these results support the hypothesis that there is an interaction of the DPA, which is coordinated with the Gd^3+^ in the Gd(DPA)_3_ complex, with the Eu^3+^, which is in the network forming the hydrogel.

Although the pre‐exponential factor of the lifetime decay curve fit cannot be used to determine the relative concentration between the pristine Eu^3+^ site of the HGEu (A_1_) and the Eu^3+^ site that interacts with the Gd(DPA)_3_ complex (A_2_), their percentage value can be used to estimate the growth/downward trend of the relative concentration. Thus, Table 2 shows a progressive increase in the A_2_ factor as the concentration of the Gd(DPA)_3_ complex increases in the HGEu‐Gd(x) hybrid; in contrast, the A_1_ factor decreases. Using direct excitation at the ion (λ_ex_ = 395 nm), this tendency is less evident due to the low molar absorptivity of the Eu^3+^ (Table S2). From this, we can infer that the concentration of the Eu^3+^ site that interacts with the Gd(DPA)_3_ complex increases, whereas the concentration of the pristine Eu^3+^ site of the HGEu decreases, as the concentration of the Gd(DPA)_3_ complex increases, reinforcing the hypothesis of the coordination of the DPA ligand to more exposed Eu^3+^ ions at the gel network.

**FIGURE 4:**
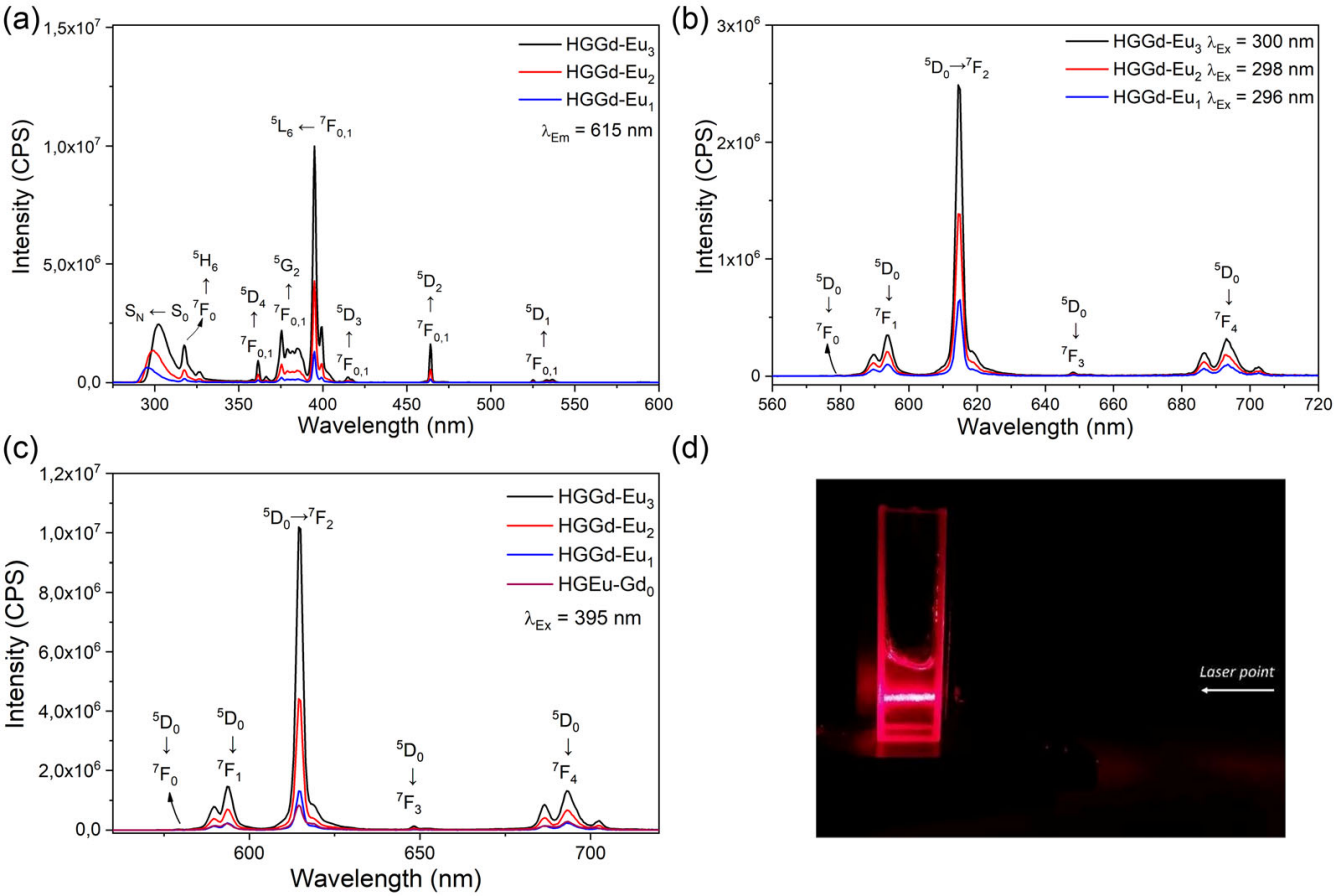
(a) Excitation spectra (λ_Em_ = 615 nm) of the HGGd‐Eu(0)‐(3) hybrids. (b) Emission spectra, under excitation at the ligand, of the HGGd‐Eu(1)‐(3) hybrids. (c) Emission spectra (λ_Ex_ = 395 nm) of the HGGd‐Eu(1)‐(3) hybrids. (d) Optical pathway of a red beam laser in the HGGd‐Eu(x) hybrid in a quartz curvet.

The HGGd‐Eu(1‐3) system was employed to investigate whether the luminescence of the complex is influenced by interactions with the gel matrix, by means of changes in the coordination environment, monitored through the luminescence behavior of the Eu^3+^ ions. The excitation spectra of the hybrid hydrogels HGGd‐Eu(1‐3) (Figure 4a) exhibit the same spectral profile as that observed for Eu(DPA)_3_ solutions (Figure S6a). Moreover, a progressive increase in the intensity of the excitation band attributed to ligand absorption is observed with increasing concentration. However, the emission intensity from the ligand excitation band in the HGGd‐Eu(1‐3) gels shows a smaller increase than the Eu(DPA)_3_ solutions, due to the light scattering effect in the gel colloidal medium, which attenuates the excitation power (Figure 4d).

The emission spectra of the hybrid hydrogels, when excited at ligand absorption region (λ_ex_ = 297, 299, and 302 nm for HGGd‐Eu(1), (2), and (3), respectively), and directly into the Eu^3+^‐4f excited state, 395 nm, are shown in Figure 4b and c, respectively. As well as HGEu‐Gd(0‐3) hybrids show linear increase in emission intensity, under excitation at 395 nm, with increasing concentration of the Gd(DPA)_3_ complex, the emission intensity of the HGGd‐Eu(1‐3) hybrids shows similar behavior; however, the intensity increases faster than HGEu‐Gd(1‐3) (Figure S9). Furthermore, the emission intensity of the hybrid hydrogels is lower than that observed for Eu(DPA)_3_ solutions, due to light scattering by the colloidal gel matrix, known as the Tyndall effect, demonstrated by the bright red light beam within the gel (Figure 4d).

The maintenance of the emission spectral profile of the Eu(DPA)_3_ complex within the gel, compared to aqueous solutions, indicates that the coordination polyhedron symmetry is unaffected by interactions with the gel fibers. Luminescence decay curves for HGGd‐Eu(1‐3), after both ion and ligand excitation with emission monitored at 615 nm (Table S3), showed a bi‐exponential decay profile, suggesting two distinct emissive sites. We hypothesized that one of them corresponds to the Eu^3+^ site in Eu(DPA)_3_ complex dispersed in this new environment, with possible supramolecular interactions, leading to a shorter lifetime than that obtained for Eu(DPA)_3_ aqueous solution (1.47 ms, Figure S3). The other site corresponds to the Eu^3+^ site of the complex with the DPA ligand coordinates also with the Gd^3+^ of the gel. This behavior, together with the reduction of lifetime and, along with the unchanged emission spectral profile, supports the hypothesis that DPA‐hydrogel fiber interactions change excited‐state deactivation dynamics, increasing non‐radiative processes. Additionally, it may suggest no replacement between Gd and Eu in the HGGd gel and Eu(DPA)_3_ complex, respectively. To verify this, a small amount of Gd(NO_3_)_3_ was solubilized in an aqueous solution of Eu(DPA)_3_ (17 mmol L^−1^). The result solution maintained a transparent aspect in contrast to the experiment with Gd(DPA)_3_ and Eu(NO_3_)_3_. The Eu^3+^‐emission spectral profile has not shown any change; however, the emission intensity has dramatically diminished (Figure S10). This behavior suggests replacement between Gd and Eu ions and reinforces the hypothesis of no ion exchange in the HGGd‐Eu(1‐3) hybrids.

As observed in the HGEu‐Gd(x) hybrids, for the HGGd‐Eu(x) the shorter lifetime (τ_1_) is attributed to Eu(DPA)_3_, which DPA coordinates to Gd^3+^ of the gel, while the longest lifetime (τ_2_) corresponds to Eu(DPA)_3_ that performs supramolecular interaction only. The increase in complex concentration introduces new molecules that behave similarly to those in aqueous solutions.

Evidence to support the saturation of interaction between Ln(DPA)_3_ complex and the hydrogel network may be accompanied by pre‐exponential factors obtained from the exponential fit of the luminescence decay curve after 395 nm excitation. For HGGd‐Eu(1‐3), the contribution of species associated with τ_1_ decreased progressively (53%, 40%, and 24%, respectively), indicating that higher complex concentrations saturate hydrogel surface interactions.

The HGGd‐Gd(x) hybrids were prepared to investigate the luminescence behavior of the gel matrix and ligand in the absence of an emissive ion. As observed for the HGGd‐Eu hybrid hydrogels, Gd^3+^ transitions could not be identified due to the strong absorption of DPA in the same region. The excitation spectrum of the HGGd‐Gd(x) hydrogels, upon monitoring emission at 410 nm (Figure S11a), displayed a broad band with three distinct features: The first, at 310 nm, corresponds to the S_1_ ← S_0_ transitions of the DPA ligand; the second, at 341 nm, is assigned to the intraligand transition of the IDA ligand [17]; and the third, at 359 nm, is the Raman signal of water, according to Equation S1.

These results indicate that the emission behavior of the organic components of the HGGd‐Gd(x) hybrids behave independently and can be switched by tuning the excitation wavelength. The progressive increase in the intensity of the excitation band at 310 nm in the HGGd‐Gd(x) hybrids is observed (Figure S11b), confirming the assignment of this band to the DPA ligand.

Thus, emission spectra of the HGGd‐Gd hydrogels were obtained by exciting both at the DPA ligand (λ_ex_ = 310 nm) and at the IDA IL band (λ_ex_ = 334 nm) states (Figure S11c), revealing predominant fluorescence from DPA at 402 nm, along with co‐fluorescence from both the DPA and IDA components, as well as the Raman signal of water.

### 2.10 Structural Features Based on Theoretical Calculations and Experimental Evidence

Molecular geometry modeling is one of the steps for an adequate prediction of the luminescence properties of a system. Thus, in this work, to understand the interactions that led to the increase in Eu^3+^ luminescence and the HGEu‐Gd hydrogels stiffness through the addition of the Gd(DPA)_3_ complex, a structural models of the HGEu‐Gd hybrid and the HGEu hydrogels systems were proposed, namely, the complexes [Eu_2_(IDA)_6_(HDPA)_2_(H_2_O)_4_]^2–^ and [Eu_2_(IDA)_6_(H_2_O)_6_], respectively. The first one was proposed based on the interaction of the HDPA ligand of the Gd^3+^‐complex with the hydrogel (for simplicity, this model considered only one ion, in this case, the Eu^3+^ ion from hydrogel, and a partially deprotonated HDPA anion coordinated to only this ion); and the last one was proposed based on the Freire et al.'s work for the metallogels with Eu(III) ion and IDA ligand. These complexes were optimized in the ground state using the semiempirical PM3 method with the Sparkle model (Sparkle/PM3) [40].

**FIGURE 5:**
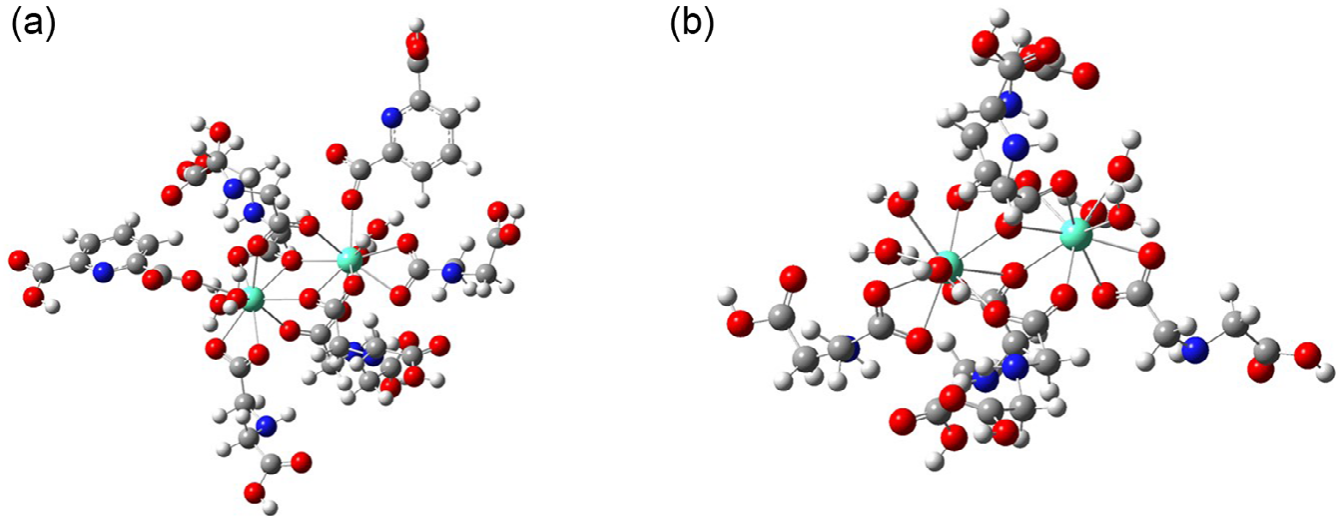
Sparkle/PM3 calculated structure of the (a) [Eu_2_(IDA)_6_(HDPA)_2_(H_2_O)_4_]^2–^ and (b) [Eu_2_(IDA)_6_(H_2_O)_6_] complexes. The spheres in green represent the europium ions, in blue the nitrogen atoms, in red the oxygen atoms, in gray the carbon atoms, and in white the hydrogen atoms.

The experimental results characterizing the photophysical properties show excellent agreement with the proposed models for obtaining theoretical luminescent properties. The proposed structural models, as well as the level of theory used for geometry optimization, were adequate for obtaining an optimal fit between the theoretically calculated Judd‐Ofelt intensity parameters and those experimentally obtained values from the emission spectra. Figure 5 shows the optimized geometry in the ground state and the coordination environment of the Eu^3+^ ions for the proposed models of HGEu‐Eu and HGEu, respectively, the complexes [Eu_2_(IDA)_6_(HDPA)_2_(H_2_O)_4_]^2–^ and [Eu_2_(IDA)_6_(H_2_O)_6_].

Both systems are homobimetallic, with two lanthanide centers of Eu^3+^ ions. The first model complex has six IDA ligands, two HDPA ligands, and four water molecules. Each Eu^3+^ ion is coordinated to nine oxygen atoms: six from IDA ligands (two bidentate, four bridge mode of coordination of the IDA ligands), one from HDPA ligand (monodentate mode), and two from water molecules. The coordination geometry of the nine oxygens around Eu^3+^ is compatible with a distorted tricapped trigonal prism (TTP), a typical geometry for this class of complexes [41].

**FIGURE 6:**
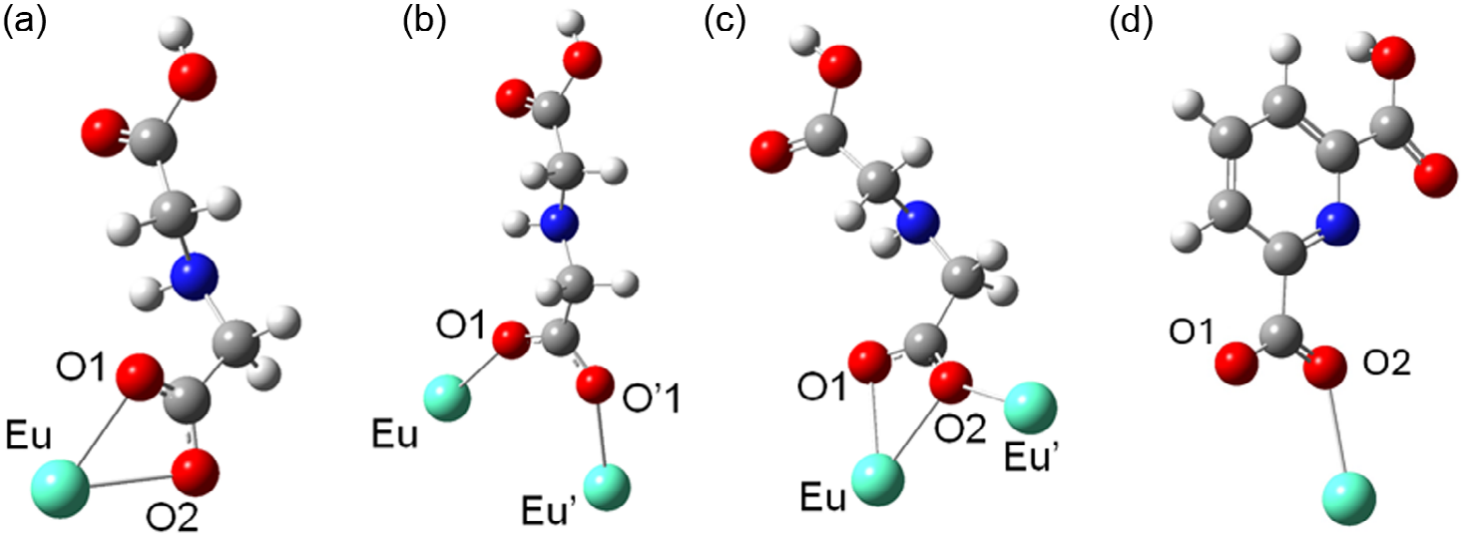
Coordination modes of the carboxylate groups of the IDA (a–c) and HDPA (d) ligands in the structure of Eu^3+^ complex models.

It is also worth highlighting the four different coordination modes adopted by the IDA ligand in the complex [Eu_2_(IDA)_6_(HDPA)_2_(H_2_O)_4_]^2–^, as illustrated in Figure 6. In coordination mode (a), the IDA anion acts as a simple bidentate chelating ligand (*syn*, *syn‐η^1^:η^1^*), coordinating to a single Eu^3+^ center through two oxygen atoms of the carboxylate group. In mode (b), the carboxylate group binds two Eu^3+^ ions through a bidentate bridge (*syn*, *syn‐η^1^:η^1^:μ^2^*). In mode (c), the carboxylic oxygens function as bidentate chelators for an Eu^3+^, and one of the oxygen atoms additionally binds to another Eu^3+^ ion, forming a monoatomic bridge or μ‐oxo bridge (*η^2^:η^1^:μ^2^*). Finally, in coordination mode (d), the HDPA anion acts as a monodentate ligand (*η^1^*), coordinating to a single Eu^3+^ center via a carboxylic oxygen. The structure of this complex preserves the structural arrangement calculated for the complex [Eu_2_(IDA)_6_(H_2_O)_6_], in which one water molecule is changed by an HDPA anion.

Taken together, the structural results obtained from geometric optimizations using the Sparkle/PM3 method, combined with experimental evidence, consistently support the proposed models. The adequate description of the coordination environment of Eu^3+^ ions, as well as the identification of the different coordination modes of the IDA and HDPA ligands, allows us to rationalize the observed modifications in the photophysical and mechanical properties of the hybrid hydrogels. Thus, the adopted structural modeling proves appropriate for representing the key interactions responsible for increasing the material's stiffness and modulating the luminescence of Eu^3+^, providing a solid basis for the interpretation of the optical properties discussed in subsequent section.

### 2.11 Intensity Parameters (Ω_j_), Triplet and Singlet Excited States, and Theoretical Modeling of Intramolecular Energy Transfers (IET) Mechanism

The Judd–Ofelt intensity parameters are highly dependent on the coordination polyhedron geometry [32]. The experimental and theoretical data obtained are shown in Table 1. An overall increase in Ω_2_ is observed as the DPA contribution is raised in the hybrid gels, whereas Ω_4_ shows the opposite overall trend. Moreover, the Ω_2_ values obtained here (5.1–6.7 × 10^−20^ cm^2^) are lower than those reported for Eu^3+^ 4‐halogenobenzoate carboxylates, while Ω_4_ remains within the range commonly reported for carboxylate‐based Eu^3+^ systems, including thiophene‐2‐carboxylate complexes [42, 43]

It is worth noting that the intensity parameters for the HGEu hydrogel (Table 1) were similar to those previously reported [19]. It is observed that the relaxation processes in HGEu are predominantly governed by nonradiative mechanisms, A_nrad_ = 3132 cm^−1^, mainly due to vibrational quenching from N–H oscillators of the ligand and, also, O–H groups from possible water molecules in the first coordination sphere of the Eu^3+^ ions, leading to poor sensitization of Eu^3+^ luminescence and a quantum efficiency of 9.2% [17].

Due to limitations in estimating the fractions of the Eu^3+^ ion in the HGEu gel network that interact with Gd(DPA)_3_ complexes and the fraction that maintains the pristine HGEu, our model of complete interactions in HGEu‐Gd, that is, no one Eu^3+^ ion without interact with DPA ligand of the Gd‐complex, was simulated using experimental data of the HGEu‐Gd(3). The theoretical values of Ω_2_ and Ω_4_ obtained for the proposed models of the hydrogel HGEu and HGEu‐Gd were close to the experimental values, which corroborates the structural proposal presented in Figure 5, and suggest there is a saturation of the DPA interaction at the higher concentration (3), since the good agreement between theoretical value from the [Eu_2_(IDA)_6_(HDPA)_2_(H_2_O)_4_]^2–^ complex and the experimental value for the HGEu‐Gd(3). Thus, our assumption of that the HGEu‐Gd(3) would model the saturated interactions between DPA ligand (HDPA anionic ligand in our model) of the Gd‐complex and hydrogel is adequate in describing these systems.

In addition to the Judd–Ofelt intensity parameters (Ω_2_ and Ω_4_), considering the ^5^D_0_ as the main emitting state, Table 1 presents the experimental and calculated values of the radiative deactivation rate (A_rad_), nonradiative deactivation rate (A_nrad_), emission lifetime (τ), quantum efficiency ratios (η), and quantum yield (q).

**FIGURE 7:**
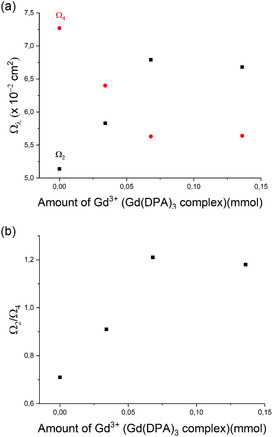
Experimental intensity parameters Ω_2_ and Ω_4_ (a) and the ratio Ω_2_/Ω_4_ (b) as a function of the amount of Gd(DPA)_3_ complex added.

Figure 7a shows the behavior of the intensity parameters Ω_2_ and Ω_4_ as a function of the amount of added Gd(DPA)_3_ complex. It can be seen that the intensity parameters show different trends; while Ω_2_ increases with the addition of the complex, Ω_4_ decreases. This trend reflects the greater influence of Ω_2_ on angular variations/distortions in the coordination polyhedron, as already mentioned through the behavior of the intensity ratio. However, a tendency toward the formation of a plateau is observed also for the ratio between the parameters Ω_2_/Ω_4_ (Figure 7b), such that for HGEu‐Gd(3) system, it seems to have already reached a plateau. This behavior corroborates the hypothesis of saturation of the possible interactions with the fiber‐forming complexes of the HGEu gel.

A significant decrease in the nonradiative decay rate (A_nrad_) and a corresponding increase in the radiative decay rate (A_rad_) with the increase of the amount of the Gd(DPA)_3_ complex added to form the hybrid HGEu‐Gd(x) systems are observed. This behavior is attributed to the progressive increase in Eu^3+^ ion sites in structures of [Eu_2_(IDA)_6_(HDPA)_2_(H_2_O)_4_]^2–^ complex type, resulting from the gradual replacement of water molecules by HDPA ligands in coordination sphere, as their concentration in the Gd(DPA)_3_ complex in the medium increases. This replacement favors the decrease in energy dissipation by high‐energy vibrations associated with water molecules and contributes to greater rigidity of the structure, through the formation of new interactions between the gel fibers, further contributing to the reduction of non‐radiative deactivation channels and promoting an increase in quantum efficiency by approximately three times from the HGEu to HGEu‐Gd [44].

The calculated singlet and triplet excited state energy values for the complexes models are shown in Table S4. As can be observed, the first triplet state for the HGEu‐Gd complex model have higher energy (~ 22,170 cm^–1^) than the main emitting state of Eu^3+^ (^5^D_0_), suggesting an efficient intramolecular energy transfer channel in these systems. In fact, the orbitals that contribute most to the formation of the triplet state are located on the HDPA ligand, with a contribution of ~ 51% (Figure S12). On the other hand, the value for the HGEu complex model is too high (> 32,300 cm^–1^), which does not favor the efficient intramolecular energy transfer, consistent with the fact that this compound is not luminescent. The level of theory used also allowed for a good agreement of the calculated absorption spectrum with the experimental one (Figures S13 and Figure S1, respectively).

**FIGURE 8:**
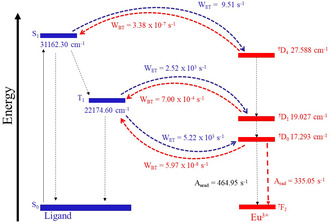
Jablonski‐type diagram of the most probable states involved in the energy transfer process of the [Eu_2_(IDA)_6_(HDPA)_2_(H_2_O)_4_]^2–^ complex (calculated from τ_ex__p_ = 1.25 ms). The solid and dashed arrows describe radiative and nonradiative processes, respectively.

The energy transfer and back‐transfer rates between the ligands and Eu^3+^ were quantified by the theoretical model developed by Malta [45] and the calculations were performed using the LUMPAC program [46, 47]. Figure 8 presents the most significant ligand‐ion transfer rates calculated. The low energy transfer rates for the model with only IDA ligands (HGEu model) suggest weak sensitization of the luminescence, which explains why the complex is not luminescent. Higher ligand to metal energy transfer rates are obtained for the Eu^3+^‐complex model with HDPA ligand (HGEu‐Gd model) (Figure 8). As the donor *T*_1_ and *S*_1_ states have energy (properly) above the acceptor Eu^3+^ ion levels, energy transfers via these states are favorable. It can be observed that there are two comparable rate transfers: via singlet state of the ligand to the ^5^D_4_ level of the ion and via triplet to the ^5^D_1_ level. Globally, the sum over all back transfer rates (ion → ligand) is much smaller than the rates of direct transfer (ligand → ion), which favors the process of sensitizing the luminescence of the europium complex.

The interpretation of luminescent properties was based on the formulation of a kinetic model that describes the temporal evolution of the populations of the most relevant electronic states in the proposed energy transfer processes. Figure 8 shows the Jablonski‐type diagram of the most probable states involved in the energy transfer process only for the [Eu_2_(IDA)_6_(HDPA)_2_(H_2_O)_4_]^2–^ complex (calculated from τ_exp_ = 1.25 ms) (model of the HGEu‐Gd(3)), since the HGEu hydrogel does not exhibit luminescence. This procedure allowed obtaining theoretical estimates for the luminescence lifetime and emission quantum yield in systems containing Eu^3+^ ions. In those proposed scenarios, the calculated quantum yield of 0.07% and 36.7%, respectively, for the proposed models of the HGEu and HGEu‐Gd(3) (calculated from τ_exp_ = 1.25 ms, related to the ligand state excitation of the Eu^3+^ site of the complex under direct influence of the DPA ligand – coordinated – experimentally determined for the HGEu‐Gd(3) hydrogel). The lifetimes were obtained from the luminescence decay curves from the ^5^D_0_ emitting state. The fit of these graphs by the exponential decay function $y=y_{0}+A_{1}exp^{-(x-x_{0})/{\text{τ}}_{1}}$, where τ_1_ corresponds to the lifetime, we obtained 0.29, and 1.24 ms, respectively, which corroborates with the experimental luminescence lifetimes.

In general, the good agreement between the Judd–Ofelt intensity parameters, radiative and nonradiative rates, lifetimes, and quantum efficiency obtained experimentally and theoretically confirms the validity of the computational model used to describe the intramolecular energy transfer processes in HGEu‐Gd hybrid hydrogels. The progressive introduction of the Gd(DPA)_3_ complex promotes a reorganization of the local Eu^3+^ environment, reducing nonradiative deactivation channels and favoring efficient sensitization mechanisms via excited states of the HDPA ligand. These results show that the saturation of ligand‐hydrogel interactions plays a central role in enhancing luminescent properties, demonstrating how the combination of theoretical modeling and experimental data is fundamental for understanding and guiding the rational design of new lanthanide‐based luminescent hybrid materials.

## 3 Conclusion

We have presented a successful experimental study and computational insights to the improvement of luminescence behavior of a supramolecular hydrogel based on the [Eu_2_(IDA)_6_(H_2_O)_6_] complex units by adding an aqueous solution of Gd(DPA)_3_ complex. The incorporation of the complex changes the absorption profile, in which the DPA ligand acts as a sensitizer of Eu‐luminescence. Furthermore, the maximum luminescence intensity increases by ~ 7 times upon direct excitation of the Eu ions. Additionally, the incorporation of the Ln(DPA)_3_ complex reinforces the hydrogel network and enhances its mechanical rigidity. This strategy demonstrated to be efficient for modifying the sensitization pathway of luminescent Ln‐gel by adding a complex with a known ligand sensitizer without luminescent ion.

Overall, the combination of experimental results and computational modeling proved essential to elucidate the structure‐property relationships in Eu^3+^‐based luminescent hybrid hydrogels. The proposed theoretical models consistently reproduce the coordination environment of lanthanide ions and explain the observed evolution of photophysical parameters, especially the increase in quantum efficiency and emission lifetimes with the incorporation of the Gd(DPA)_3_ complex. The progressive substitution of water molecules by DPA ligands in the Eu^3+^ environment significantly reduces non‐radiative deactivation channels and favors efficient intramolecular energy transfer mechanisms, consistent with Judd–Ofelt parameters and kinetic calculations. These findings reinforce the role of coordination environment engineering as a key strategy for the rational development of lanthanide‐based hybrid materials, opening perspectives for the optimization of luminescent systems in soft matrices with potential application in optical devices and biofunctional systems.

## 4 Experimental Section

### 4.1 Materials

The reagents used in this study were: Gd_2_O_3_ 99.95%, iminodiacetic acid (H_2_IDA), dipicolinic acid (H_2_DPA) 99,9%, sodium hydroxide, and Eu_2_O_3_, all purchased from Sigma–Aldrich. Ethanol (99.8%, analytical grade) and nitric acid (65%, analytical grade) were obtained from NEON. The lanthanide nitrates, Ln(NO_3_)_3_·6H_2_O (Ln = Eu^3+^ and Gd^3+^), were prepared by reacting the respective oxides with 65% nitric acid.

### 4.2 Synthesis of Complexes de Na_3_[Ln(DPA)_3_]·9H_2_O (Ln = Eu e Gd)

The synthesis of the complexes Na_3_[Ln(DPA)_3_].9H_2_O = Ln(DPA)_3_ (Ln = Gd^3+^ and Eu^3+^; DPA = dipicolinate anion) was performed using lanthanide nitrates, following protocols described in the literature [28, 34].

**FIGURE 9:**
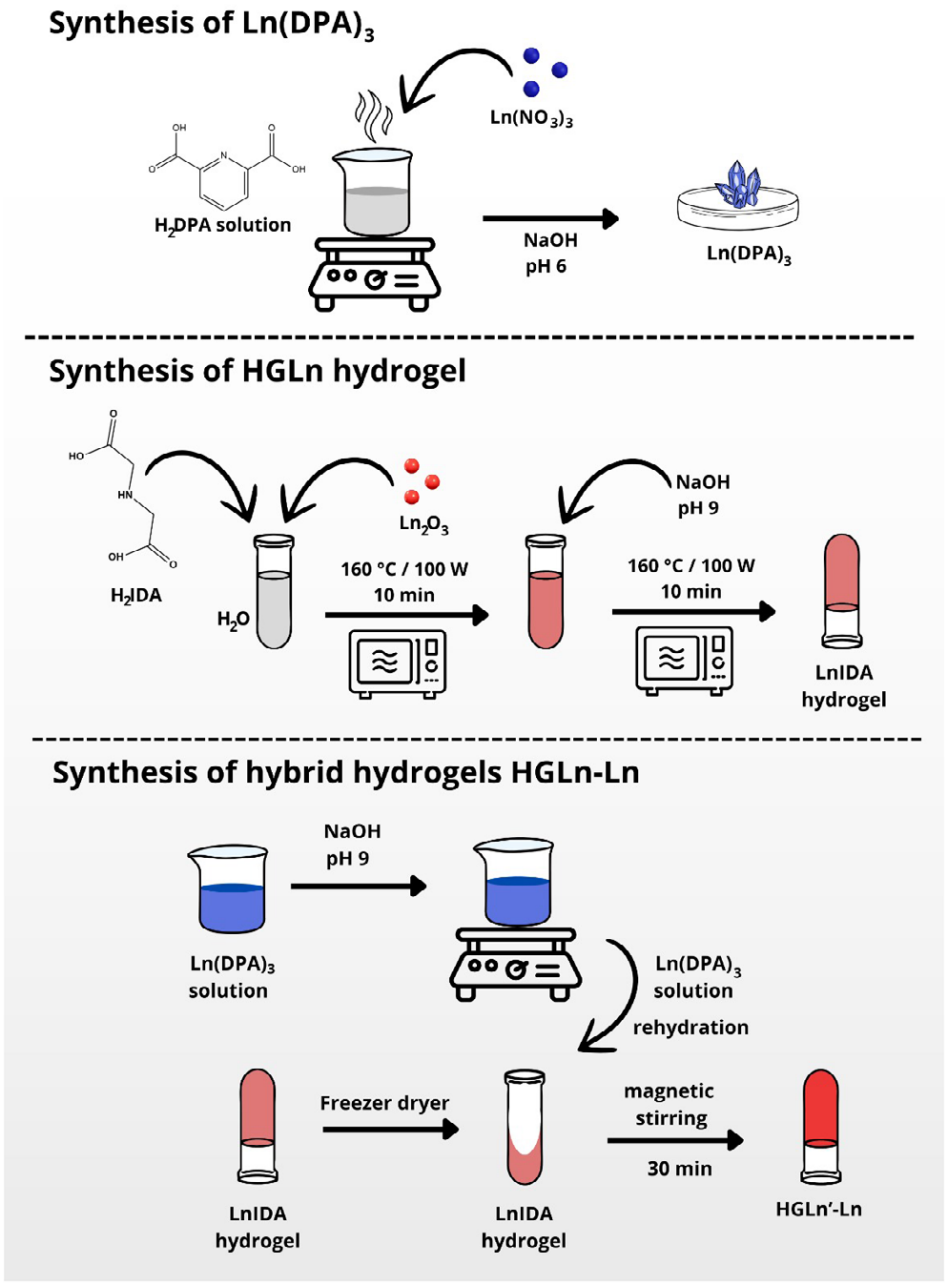
Schematic representation of the synthesis approach of Ln(DPA)_3_ complex: LnIDA hydrogel and hybrid hydrogel HGLn‐Ln (Ln = Eu^3+^ and Gd^3+^) according to above mentioned methodology.

In the adopted procedure, 3.00 mmol of dipicolinic acid (H_2_DPA) were dissolved in 50 mL of boiling water, followed by the addition of 1.00 mmol of Ln(NO_3_)_3_.6H_2_O (Figure 9). Subsequently, sodium hydroxide solution (1 mol·L^−1^) was added to adjust the pH to 6. The resulting solution was left undisturbed until complete solvent evaporation. The solid obtained for each complex was recrystallized from a water/ethanol mixture (1:2).

### 4.3 Synthesis of Lanthanide Hydrogels, HGLn

The lanthanide hydrogels HGLn (Ln = Eu and Gd) were prepared in two steps using a microwave‐assisted hydrothermal method, as reported in prior research [17, 18]. In the first step, 0.45 mmol of iminodiacetic acid (H_2_IDA) and 0.075 mmol of Ln_2_O_3_ (Ln = Eu and Gd) were added to a glass reactor containing 4 mL of ultrapure water. This suspension was then heated in a CEM microwave at 160°C (maximum power of 100 W) for 10 min under high magnetic stirring. At the end of this step, a clear and colorless solution was obtained. In the second step, the pH of the solution obtained in step 1 was adjusted to 9 using a NaOH solution (1 mol·L^−1^). Gelation was then triggered by microwave heating at 160°C (maximum power of 100 W) for 10 min (Figure 9).

### 4.4 Preparation of Hybrid Gels HGLn‐Ln(x)

The following systems were prepared: Na_3_[Gd(DPA)_3_]·9H_2_O complex incorporated into europium hydrogel (HGEu‐Gd), Na_3_[Eu(DPA)_3_]·9H_2_O complex incorporated into gadolinium hydrogel (HGGd‐Eu), and Na_3_[Gd(DPA)_3_]·9H_2_O complex incorporated into gadolinium hydrogel (HGGd‐Gd). The amount of the Ln(DPA)_3_ complexes added is presented in Table 3.

**TABLE 3:** Lanthanide ions molar relation in the HGLn’‐Ln hybrids.

Hybrid	Ln’:Ln	Ln, mmol	**Na**_**3**_ **[Ln(DPA)**_**3**_ **]·9H**_**2**_ **O, mol L** ^**−1**^
HGLn’‐Ln (0)	1:0	0	0
HGLn’‐Ln (1)	1:0.23	0.034	0.017
HGLn’‐Ln (2)	1:0.45	0.068	0.034
HGLn’‐Ln (3)	1:0.91	0.136	0.068

For the preparation of the hybrid hydrogels HGLn‐Ln, the respective aqueous solutions of the Ln(DPA)_3_ complexes were added to vials containing the partially dried hydrogels (HGLn). The solutions of the complex were prepared by dissolving the powders in water, with the pH adjusted to 9 using NaOH solution (1 mol·L^−1^). After adding the solution of the complex to the vial containing the partially dried hydrogels, the volume was adjusted with ultrapure water, when necessary, and the samples were stirred magnetically for 30 min. Any characterization of the resulting hybrid hydrogels was performed only after at least 2 h of the synthesis procedure. A control sample, HGLn‐Ln(0) (Ln = Eu and/or Gd), was also prepared by rehydrating only with ultrapure water after freeze‐drying.

Partial solvent removal was achieved by freeze‐drying 2.0 mL of hydrogel in a 5.0 mL cryogenic tube for 2.5 h.

### 4.5 Characterization Methods

Fourier transform infrared spectra (FT‐IR) were recorded from solid samples of the compounds in KBr pellets (4000–400 cm^−1^) using the Agilent Cary 600 Series FTIR spectrophotometer (model 660) with an attenuated total reflectance (ATR) accessory, resolution of 2 cm^−1^. The number of crystallization water molecules in the complexes was determined by thermogravimetric analysis (TGA), acquired in a Shimadzu TGA‐60/60H instrument. The analyses were carried out up to 900°C in a platinum sample holder, under a synthetic air atmosphere at a flow rate of 100 mL·min^−1^ and a heating rate of 10°C·min^−1^. SEM images were acquired using a TESCAN MIRA 3 microscope at 10 kV. Samples were frozen at −60°C, lyophilized, and sputter‐coated with gold before imaging.

Rheometry tests were performed using an Anton Paar rheometer (model MCR 102) equipped with a circular stainless steel parallel plate geometry with a diameter of 25 mm (PP25) and employing a plate separation gap of 1 mm. Temperature control was established with a Peltier system connected to the bottom plate. The temperature was set at 25 C for all experiments. Dynamic oscillatory rheological measurements of the hydrogels and hybrid hydrogels were performed in frequency mode. For the latter mode, the strain amplitude was fixed at 1%, within the linear viscoelastic region. The oscillation frequency (ω) was varied from 0.1 to 100 rad/s, and 15 points where recored per experimental run. For each experimental point, both storage (G’) and loss (G’’) moduli were recorded.

UV‐visible absorption spectra of the complex solutions were recorded using a PerkinElmer spectrophotometer (Lambda 60 model), with a quartz cuvette of 1 cm optical path length. Luminescence measurements (excitation and emission spectra, as well as luminescence decay curves) were performed in a Horiba Jobin‐Yvon Fluorolog‐3 spectrofluorometer equipped with continuous (450 W) and pulsed (150 W) xenon lamps as excitation sources and a Hamamatsu R928P photomultiplier tube as the detector. The emission was corrected for the spectral response of the emission monochromators and the detector using a typical correction spectrum provided by the manufacturer. The decay lifetimes were obtained by fitting decay curves with equation I(t) = I_0_ + A_1_*exp(‐t/τ_1_) (for Eu(DPA)_3_ and HGEu materials) and with equation I = I_0_ + A_1_*exp(‐t/τ_1_) + A_2_*exp(‐t/τ_2_) (for hybrid materials). In these equations, I_0_ is the emission intensity at *t* = 0 and τ is the decay lifetime. The average lifetimes (***τ***_aver._) were determined according to the following equation: $\tau_{\mathrm{Aver}.}=\frac{\sum_{i=1}^{2}A_{i}\tau_{i}^{2}}{\sum_{i=1}^{2}A_{i}\tau_{i}}$.

### 4.6 Computational Procedures

#### 4.6.1 Ground‐State Geometry Optimization and Excited States Calculation

The ground state geometry optimization for all lanthanide complex models studied was performed by the semiempirical method PM3 with the Sparkle model, Sparkle/PM3, since this combined method has an excellent capability of geometry prediction and also a considerably low computational cost [48]. The energy of the singlet and triplet excited states were calculated by using methods based on the semiempirical INDO/S method [49] implemented in Orca program [50].

#### 4.6.2 Intensity Parameters (Ω_λ_), Intramolecular Energy Transfer (IET) Rates, Kinetic Model Solution, Quantum Yield, and Emission Lifetime

The intensity parameters, Ω_λ_^theo^ (λ = 2 and 4), are obtained from emission spectra and calculated by Judd–Ofelt theory [51, 52]. The theoretical model used to calculate the energy transfer rate between the organic ligands and the lanthanide ion was developed by Malta [45]. The numerical solution of the proposed kinetic model allows calculating the quantum yield and the emission lifetime. Theoretical foundations of the models implemented in LUMPAC can be found in reference (https://lumpac.pro.br/lumpac_theory/) [46].

## Supporting Information

Additional supporting information can be found online in the Supporting Information section.

## Author Contributions

**Caroliny O. Cavalcante**: investigation, methodology, writing ‐ original draft, validation, formal analysis. **Suelen C. F. Pereira**: data acquisition, writing, formal analysis, methodology. **Sanderson H. S. Malta**: software, investigation, writing ‐ original draft and writing ‐ review & editing. **José Y. R. Silva**: conceptualization, methodology, supervision and writing ‐ review & editing. **Wilson B. Jr**: methodology and supervision ‐ review & editing. **Leonis Lourenço da Luz**: conceptualization, methodology, investigation, supervision, writing ‐ review & editing. **Juliana A. B. da Silva**: supervision, writing ‐ review & editing. **Severino Alves Júnior**: supervision, writing ‐ review & editing, funding acquisition and project administration.

## Funding

This study was supported by the Coordenação de Aperfeiçoamento de Pessoal de Nível Superior (001), the Conselho Nacional de Desenvolvimento Científico e Tecnológico (405460/2022‐8, 406820/2023‐6, 303222/2024‐7), and the Fundação de Amparo à Ciência e Tecnologia do Estado de Pernambuco (APQ‐ 0675‐1.06/14, APQ‐0549‐1.06/17, APQ‐0724‐1.06/19, APQ‐0436‐1.06/22, APQ‐1056‐1.06/22, APQ‐0677‐1.06/24).

## Conflicts of Interest

The authors declare no conflicts of interest.

## Supporting information

Supplementary Material

## Data Availability

The data that support the findings of this study are available from the corresponding author upon reasonable request.
