# Supplementary material for: Synergistic Photophysical and Mechanical Enhancement in Europium Supramolecular Hydrogel by Incorporating Gd(DPA)3 Complex (DPA = Dipicolinate)
Source: ChemistryOpen. 2026 Apr 23;15(5):e70202. doi: 10.1002/open.70202 (PMC13106984; doi:10.1002/open.70202)
Supplement: Supplementary file 1 — Supplementary Material [file OPEN-15-e70202-s001.pdf]

Synergistic Photophysical and Mechanical Enhancement in Europium Supramolecular Hydrogel by incorporating $Gd(DPA)_3$ Complex

Caroliny O. Cavalcante,[a] Suelen C. F. Pereira,[a] Sanderson H. S. Malta,[a] José Y. R. Silva,[a] Wilson B.Jr,[b] Leonis L. da Luz,*[c] Juliana A. B. da Silva*[c] and Severino Alves Júnior *[a]

[a] C. O. Cavalcante, S. C. F. Pereira, S. H. S. Malta, Dr. J. Y. R. Silva, Prof Dr. S.Alves Júnior
Departamento de Química Fundamental
Universidade Federal de Pernambuco,
Av. Prof. Moraes Rego, 1235, Cidade Universitária, Recife, Pernambuco, 50670-901, Brasil
E-mail: severino.alvesjr@ufpe.br

[b] Prof Dr. W. B.Jr
Departamento de Física
Universidade Federal de Pernambuco
Av. Prof. Moraes Rego, 1235, Cidade Universitária, Recife, Pernambuco, 50670-901, Brasil

[c] Prof Dra. J. A. B. da Silva and Dr. L. L. da Luz
Núcleo Interdisciplinar de Ciências Exatas e da Natureza
Universidade Federal de Pernambuco
Nova Caruaru, Caruaru, PE, 55.014-900, Brasil
E-mail: juliana.bsilva@ufpe.br (J.A.B.S.)/ leonisll@yahoo.com.br; leonis.lluz@ufpe.br (L. L. L.).

WILEY-VCH

Supporting Information

Table S1. Lifetime (τ_1) obtained from exponential fitting of luminescence decay curves for the $Eu(DPA)_3$ solutions.

System	λ_{ex} (nm) ligand	τ (ms)	λ_{ex} (nm) ion	τ (ms)
**$Eu(DPA)_3$(1)**	297	297	1,48	395
**$Eu(DPA)_3$(2)**	299	299	1,47	395
**$Eu(DPA)_3$(3)**	302	302	1,45	395

Table S2. Luminescence lifetime (τ_i), pre-exponential factors (A_i) and average lifetime (τ_m) obtained from exponential fitting of luminescence decay curves for the HGEu-Gd(x) hybrids.

System	Direct ion excitation					
	λ_{ex} (nm)	A_1(%)	τ_1 (ms)	A_2(%)	τ_2 (ms)	τ_m
**HGEu-Gd(1)**	395	24.8	0.17	75.2	0.54	0.448
**HGEu-Gd(2)**	395	41.8	0.26	58.2	1.09	0.743
**HGEu-Gd(3)**	395	30.2	0.24	69.8	1.19	0.903

Table S3. Lifetime (τ) obtained from exponential fitting of luminescence decay curves for the $Eu(DPA)_3$ solutions.

WILEY • VCH

System	Ligand excitation					Direct ion excitation				
	λ_{ex}(nm)	A_1	τ_1 (ms)	A_2	τ_2 (ms)	λ_{ex}(nm)	A_1	τ_1 (ms)	A_2	τ_2 (ms)
**GGd-Eu(1)**	296	61,773	0,28	85,542	1,15	395	22,690	0,27	20,239	0,96
**GGd-Eu(2)**	298	55,114	0,24	149,934	1,15	395	45,275	0,30	66,139	1,12
**GGd-Eu(3)**	301	43,641	0,21	195,032	1,24	395	63,983	0,22	199,813	1,16

Table S4. Singlet and triplet state energy associated with the Eu(III) systems and acceptor-donor distance (R_L) calculated by computational methods.

**Material**	**Singlet (cm^{-1})**	**RL (Å)**	**Triplet (cm^{-1})**	**RL (Å)**
$[Eu_2(IDA)_6(H_2O)_6]$ HGEu model	35584.30	6.2128	32340.20	6.5111
$[Eu_2(IDA)_6(HDPA)_2(H_2O)_4]^{2-}$ HGEu-Gd model	31162.30	10.2613	22174.60	10.5994

WILEY-VCH

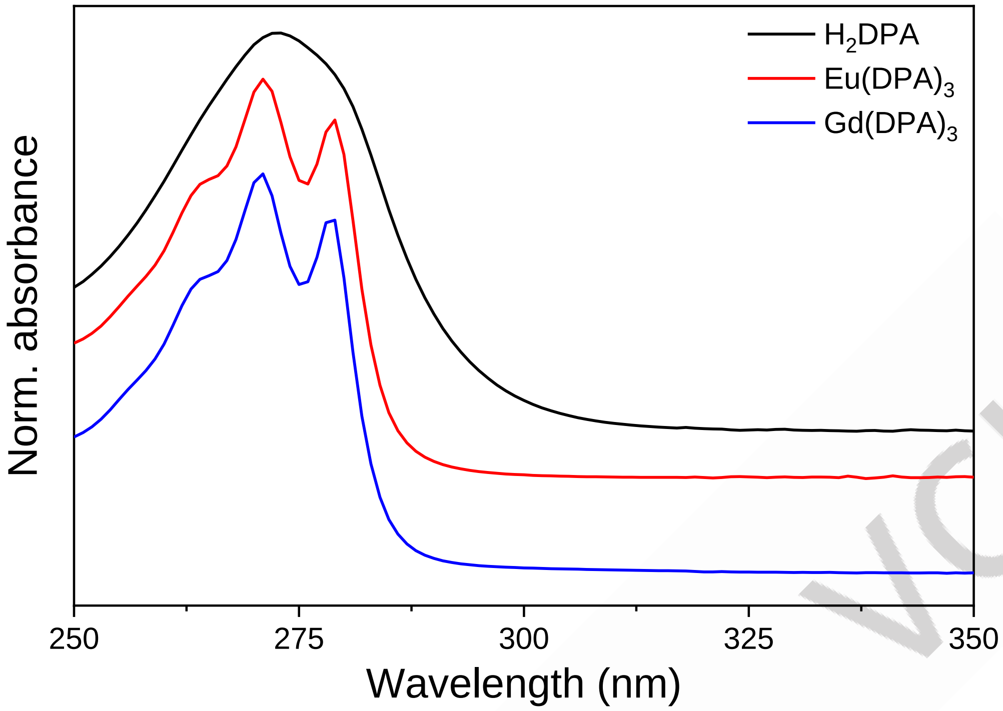

Figure S1. UV-vis absorption spectra of aqueous solutions of the $Ln(DPA)_3$ complexes and of the ligand H_2DPA

WILEY-VCH

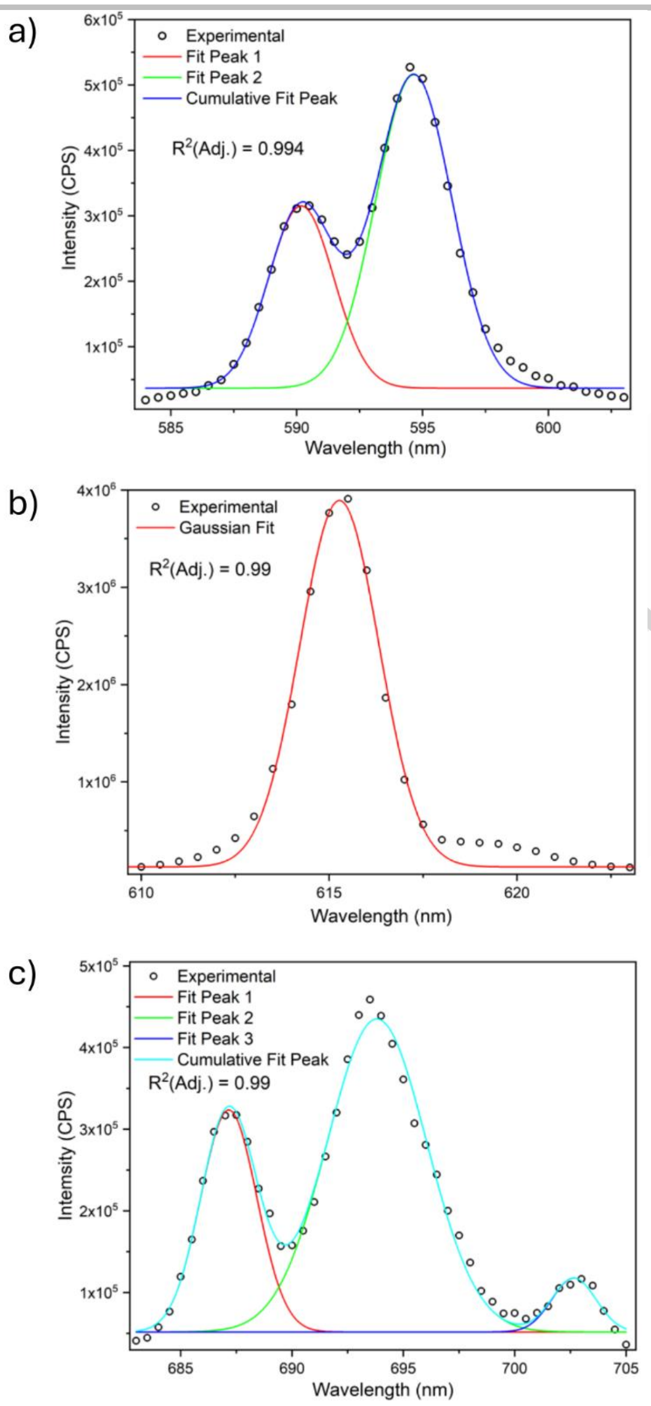

Figure S2. Deconvolution in Gaussian bands of the $^5D_0 \rightarrow {}^7F_1$(a), $^7F_2$(b), $^7F_4$(c) transitions for the emission spectrum of the Eu(DPA)$_3$ complex.

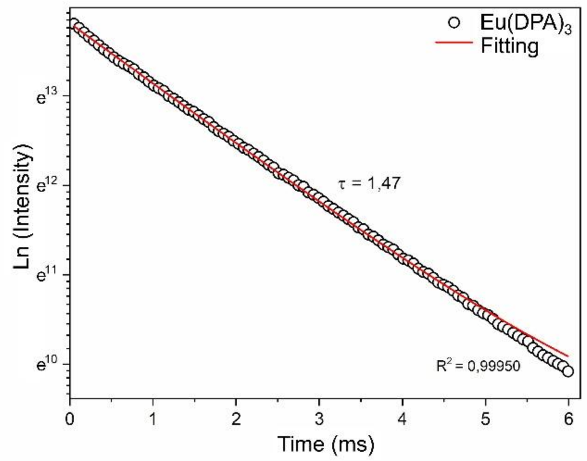

Figure S3. Emission decay curves of the $Eu(DPA)_3$ complex obtained after excitation at 300 nm and by monitoring emission at 615 nm.

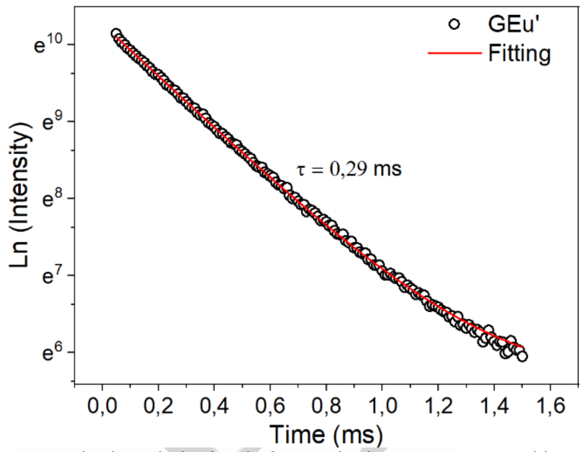

Figure S4. Emission decay curve of the EuIDA hydrogel obtained after excitation at 395 nm and by monitoring emission at 615 nm).

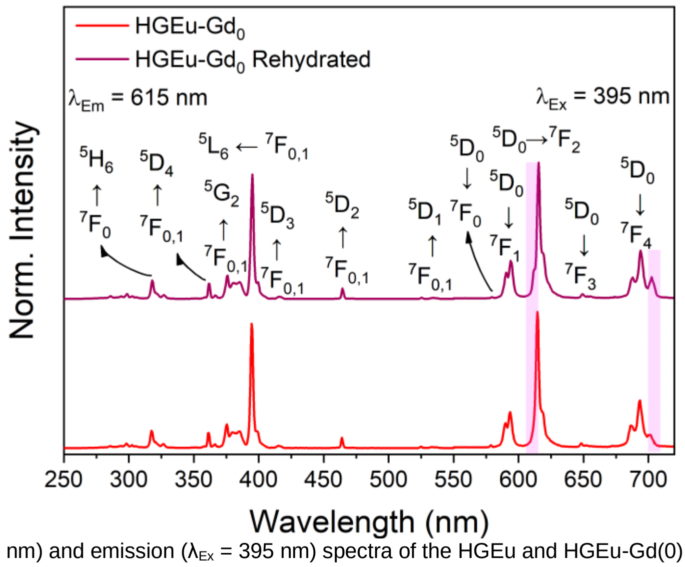

Figure S5. Excitation (λ_{Em} = 615 nm) and emission (λ_{Ex} = 395 nm) spectra of the HGEu and HGEu-Gd(0) hydrogels.

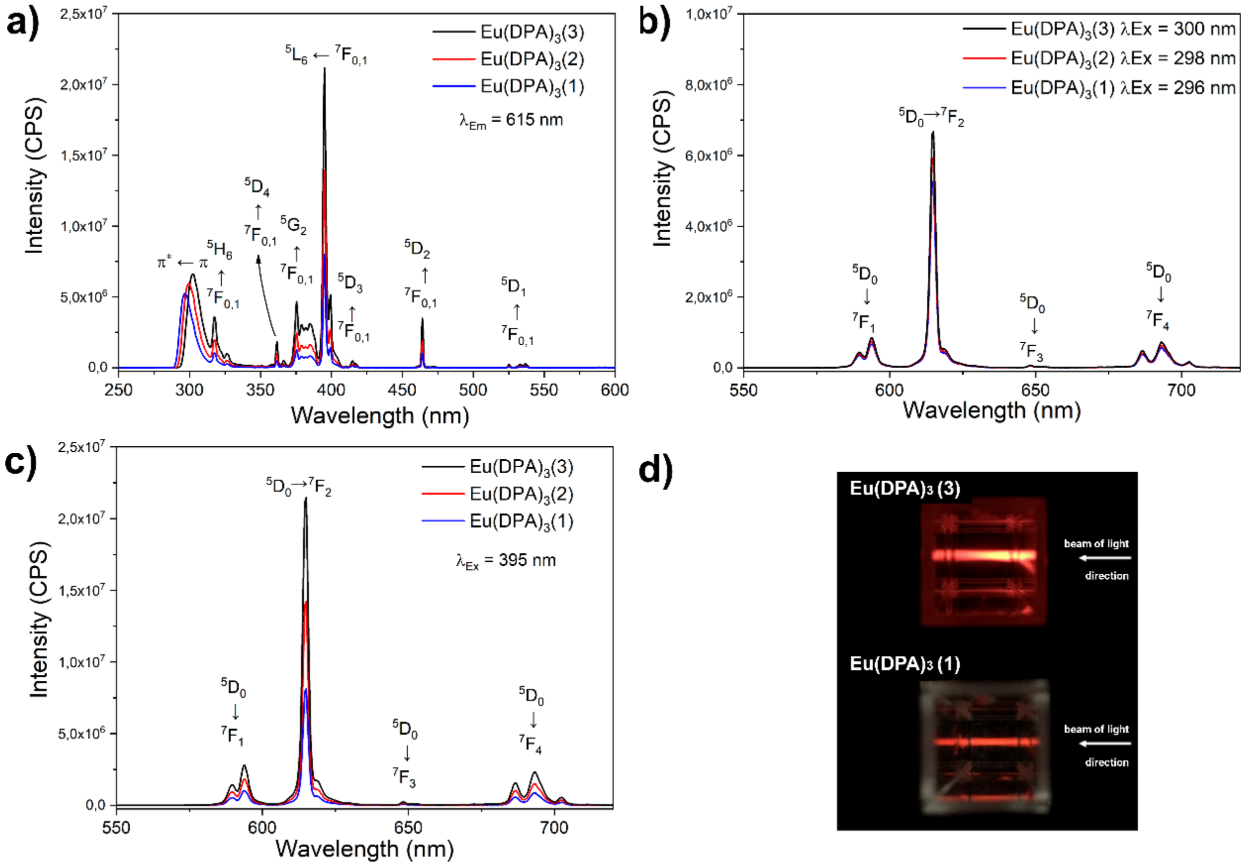

Figure S6. Photoluminescence of aqueous solution of the $Eu(DPA)_3$ complex with same concentrations of the $Ln(DPA)_3$ complex in the HGLn-Ln gels: **a)** Excitation spectra (λ_{Em} = 615 nm), **b)** emission spectra upon excitation at the ligand (λ_{Ex} = 300, 298 e 296 nm), **c)** emission spectra upon direct excitation at the ion (λ_{Ex} = 395 nm) and **d)** image of the luminescence of $Eu(DPA)_3$ solution (λ_{Ex} = 302 nm) along the optical pathway in the quartz curvet.

WILEY

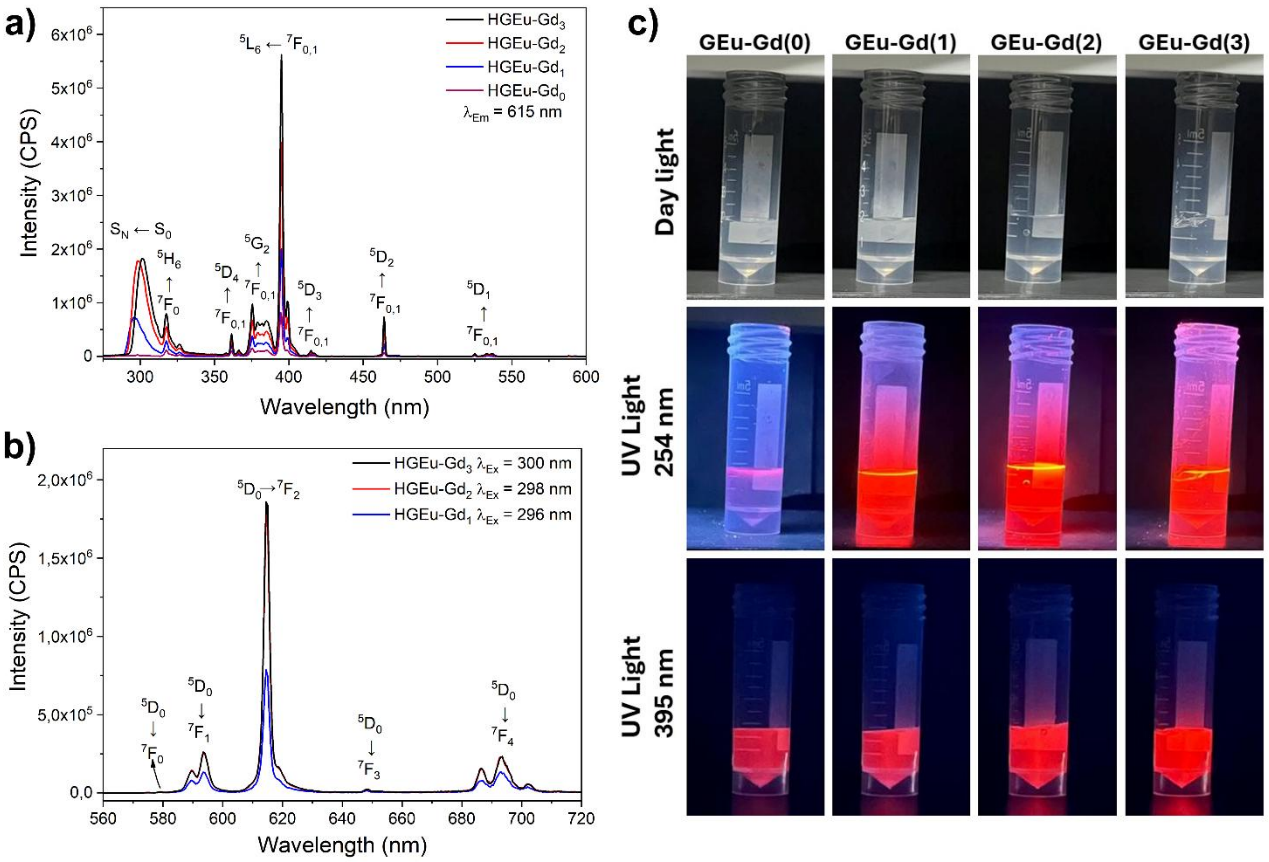

Figure S7. a) Excitation spectra (λ_{Em} = 615 nm) of the HGEu-Gd (0-3) hybrids and **b)** emission spectra (λ_{Ex} = 296, 298, and 300 nm) of the HGEu-Gd (1-3) hybrid;**c)** Photographs of the GEu-Gd(0–3) gels under daylight and under UV irradiation (254 nm and 395 nm).

WILEY

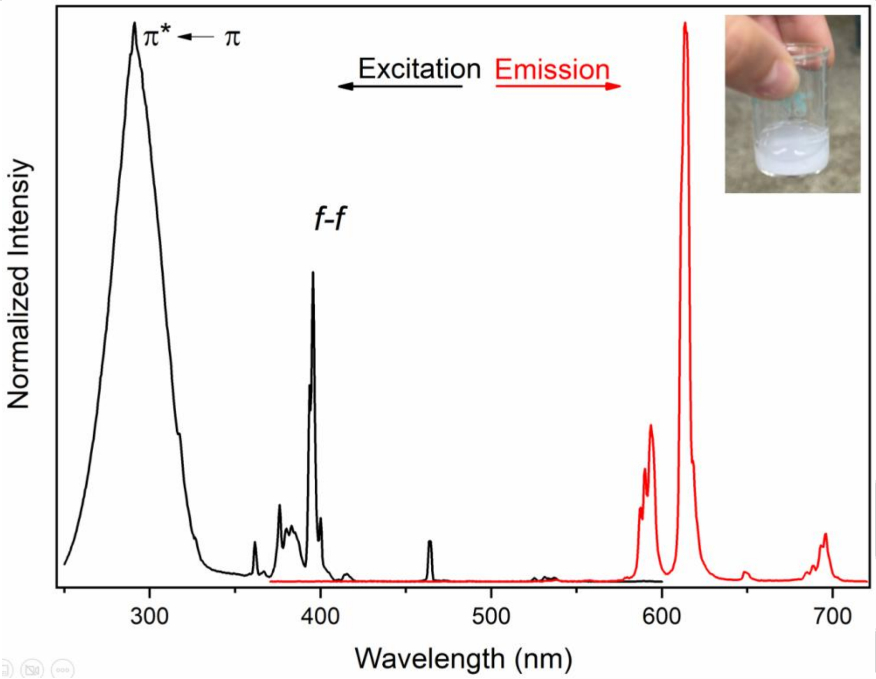

Figure S8. Excitation (λ_{Em} = 613 nm) and emission (λ_{Ex} = 290 nm) spectra of the precipitate after mixing $Eu(NO_3)_3$ and $Gd(DPA)_3$ aqueous solution.

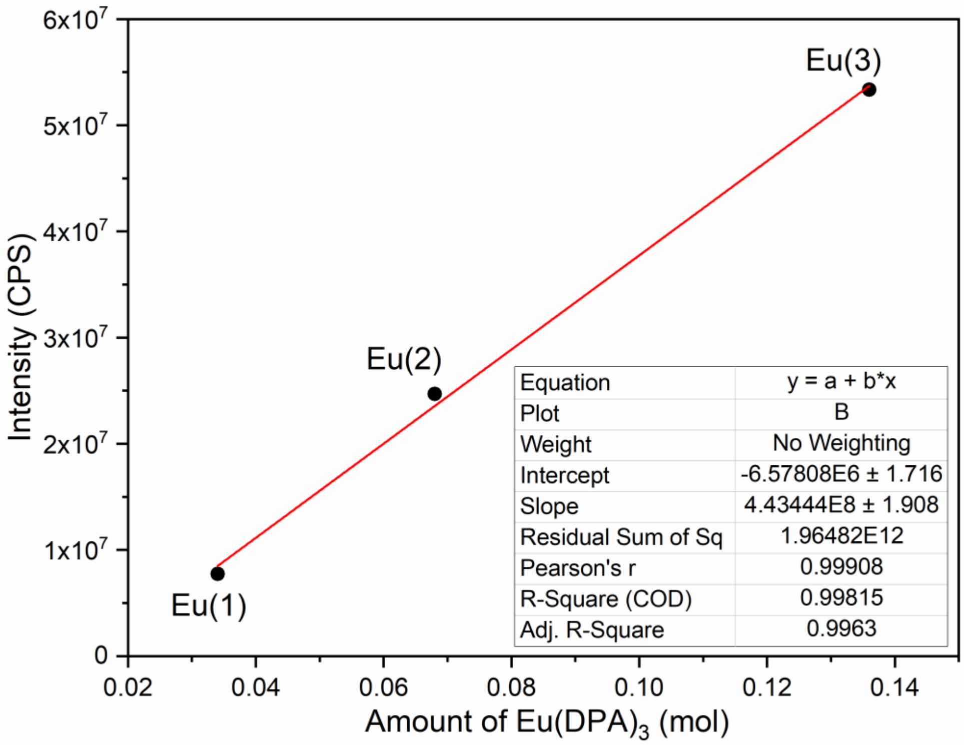

Figure S9. Integrated intensity of the emission spectra (575-720 nm) of the HGGd-Eu(0-3) hybrids as a function of the amount of the $Eu(DPA)_3$ complex.

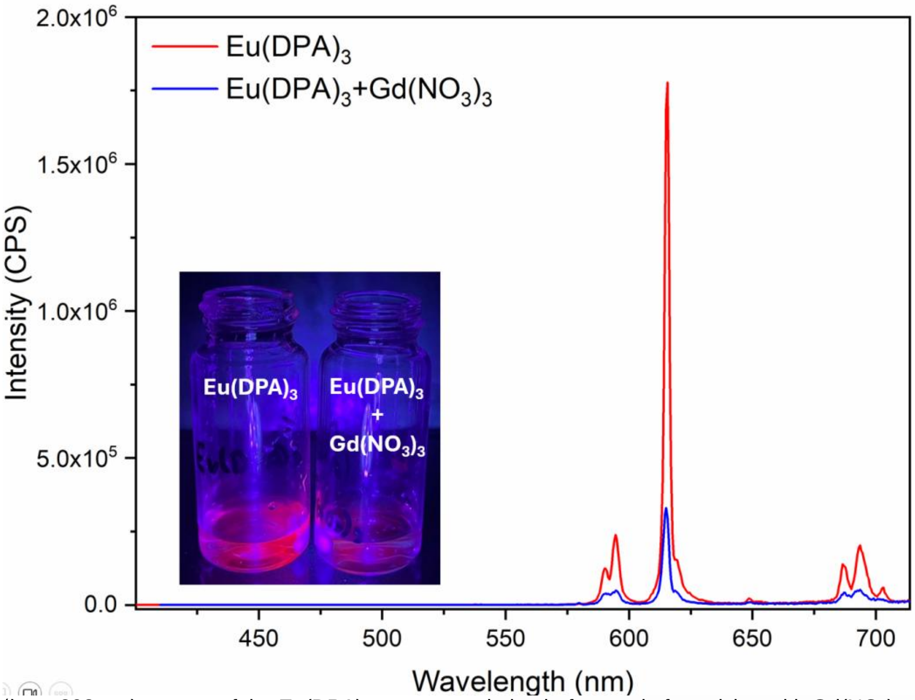

Figure S10. Emission (λ_{Ex} = 293 nm) spectra of the $Eu(DPA)_3$ aqueous solution before and after mixing with $Gd(NO_3)_3$.

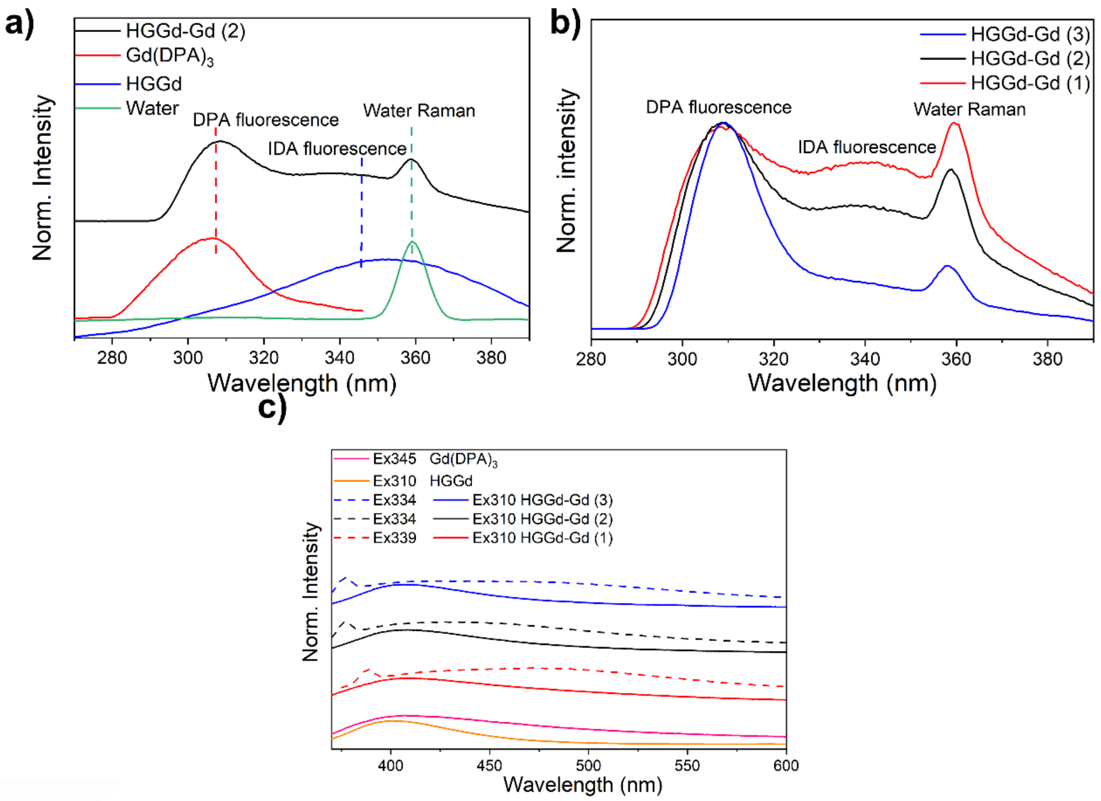

Figure S11. Excitation spectra (λ_{Em} = 410 nm) of the **a)** $Gd(DPA)_3$ aqueous solution, HGGd gel, water and HGGd-Gd(2) hybrid; **b)** HGGd-Gd(1-3) hybrid. **c)** Emission spectra (λ_{Ex} = 296, 298, and 300 nm) or the HGEu-Gd(1-3).

Equation S1.

$$\frac{1}{\lambda_{\mathrm{incident}}} = \frac{1}{\lambda_{scattered}} - \bar{\upsilon}$$

Where $\lambda_{incident}$ is the wavelength of the Raman signal, $\lambda_{scattered}$ is the wavelength of excitation and, $\bar{\upsilon}$ is the Raman shift, found between 3400-3600 cm^{-1} for water. In this work, the excitation was at 410 nm ($\lambda_{scattered}$) and the peak observed was at 359 nm, then $\bar{\upsilon}$= 3405 cm^{-1}.

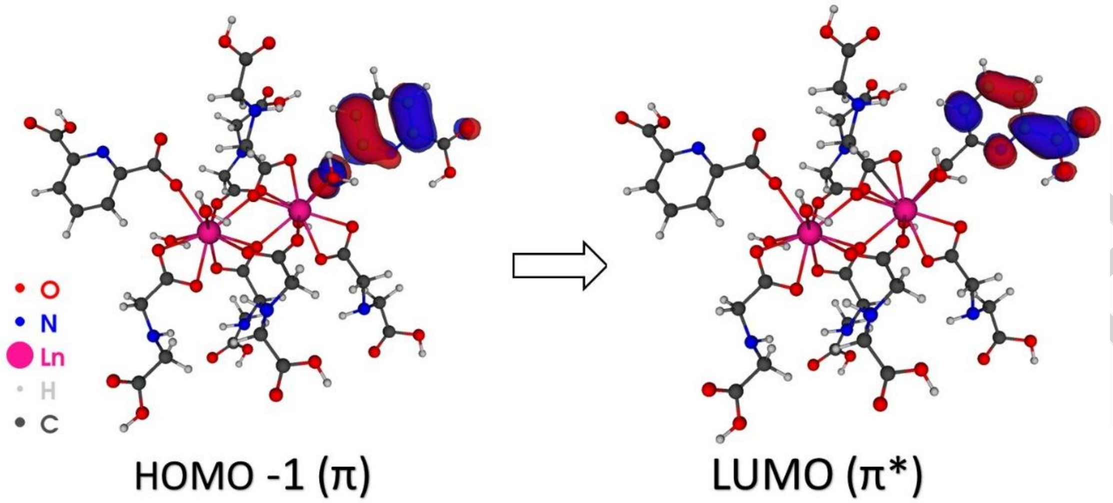

Figure S12. Representation of the electronic density of the orbitals that contribute most to the triplet energy for the system $[Eu_2(IDA)_6(HDPA)_2(H_2O)_4]^{2-}$, HGEu-Gd model, with a contribution of 50.78%.

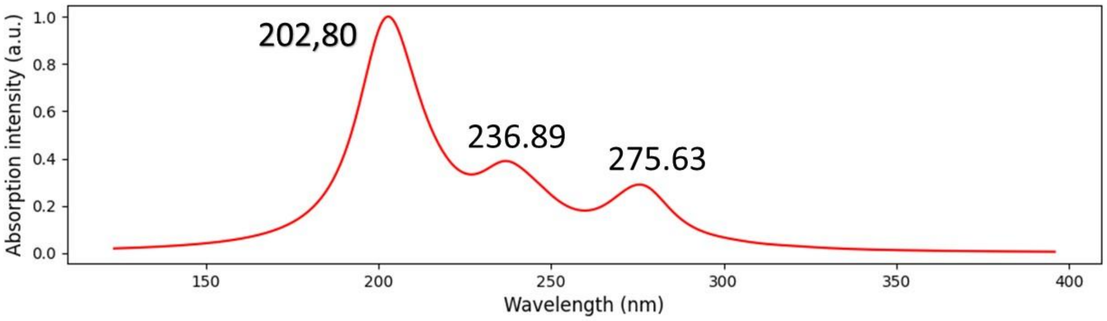

Figure S13. Simulated absorption spectrum for the system $[Eu_2(IDA)_6(HDPA)_2(H_2O)_4]^{2-}$, HGEu-Gd model, using the INDO/CIS method.

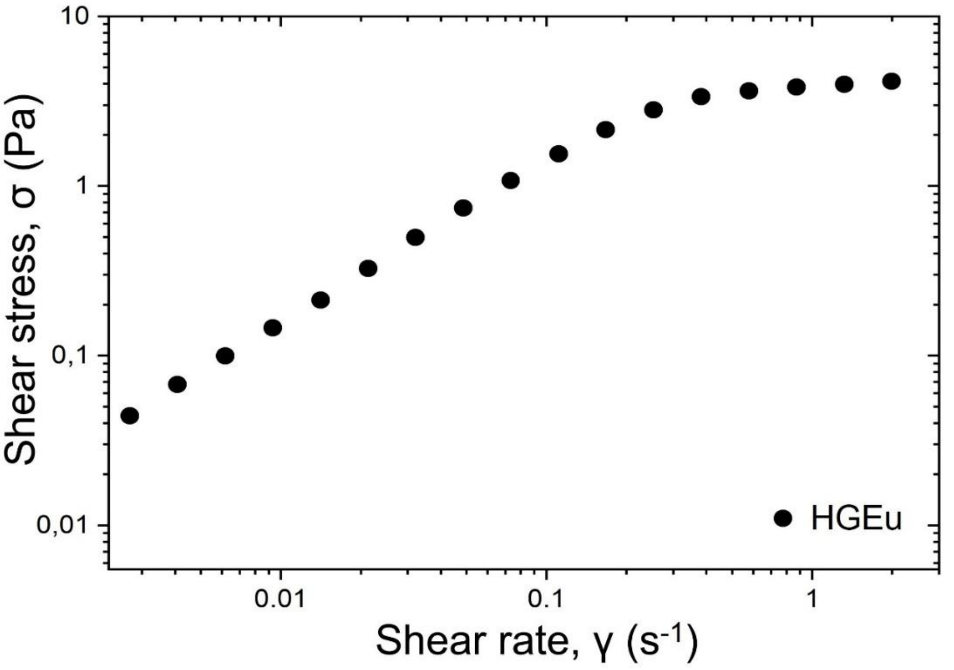

Figure S14. Shear rate sweep test of the HGE hydrogel

WILEY-VCH
